# *Cis*-acting variation is common across regulatory layers but is often buffered during embryonic development

**DOI:** 10.1101/gr.266338.120

**Published:** 2021-02

**Authors:** Swann Floc'hlay, Emily S. Wong, Bingqing Zhao, Rebecca R. Viales, Morgane Thomas-Chollier, Denis Thieffry, David A. Garfield, Eileen E.M. Furlong

**Affiliations:** 1 Institut de Biologie de l'ENS (IBENS), École Normale Supérieure, CNRS, INSERM, Université PSL, 75005 Paris, France;; 2 Molecular, Structural and Computational Biology Division, Victor Chang Cardiac Research Institute, Darlinghurst, New South Wales 2010, Australia;; 3 School of Biotechnology and Biomolecular Sciences, UNSW Sydney, Kensington, New South Wales 2052, Australia;; 4 European Molecular Biology Laboratory (EMBL), Genome Biology Unit, D-69117 Heidelberg, Germany;; 5 Institut Universitaire de France (IUF), 75005 Paris, France

## Abstract

Precise patterns of gene expression are driven by interactions between transcription factors, regulatory DNA sequences, and chromatin. How DNA mutations affecting any one of these regulatory “layers” are buffered or propagated to gene expression remains unclear. To address this, we quantified allele-specific changes in chromatin accessibility, histone modifications, and gene expression in F1 embryos generated from eight *Drosophila* crosses at three embryonic stages, yielding a comprehensive data set of 240 samples spanning multiple regulatory layers. Genetic variation (allelic imbalance) impacts gene expression more frequently than chromatin features, with metabolic and environmental response genes being most often affected. Allelic imbalance in *cis*-regulatory elements (enhancers) is common and highly heritable, yet its functional impact does not generally propagate to gene expression. When it does, genetic variation impacts RNA levels through two alternative mechanisms involving either H3K4me3 or chromatin accessibility and H3K27ac. Changes in RNA are more predictive of variation in H3K4me3 than vice versa, suggesting a role for H3K4me3 downstream from transcription. The impact of a substantial proportion of genetic variation is consistent across embryonic stages, with 50% of allelic imbalanced features at one stage being also imbalanced at subsequent developmental stages. Crucially, buffering, as well as the magnitude and evolutionary impact of genetic variants, is influenced by regulatory complexity (i.e., number of enhancers regulating a gene), with transcription factors being most robust to *cis*-acting, but most influenced by *trans*-acting, variation.

The development of a multicellular organism requires tight regulation of gene expression in both space and time to ensure that reproducible phenotypes are obtained across individuals and environmental conditions. DNA regulatory elements (e.g., promoters and enhancers) are essential to this process by integrating regulatory information from sequence-specific transcription factors (TFs), RNA polymerase II (Pol II), and other regulatory proteins to drive specific spatiotemporal patterns of expression during development. But although gene expression patterns are typically quite precise, the DNA regulatory elements that control these patterns are replete with genetic variation (mutations), which can impact transcriptional regulation at multiple levels, including TF binding (Kasowski et al. 2010; Spivakov et al. 2012; Behera et al. 2018), chromatin state (Waszak et al. 2015), transcriptional start site (TSS) usage (Schor et al. 2017), gene expression levels (Garfield et al. 2013; Battle et al. 2015; Cannavò et al. 2017), and transcript isoform diversity (Cannavò et al. 2017).

Although regulatory mutations can have large effects, many behave effectively neutrally, making predictions of the functional impact of genetic variants extremely challenging. Part of the difficulty comes from a general lack of knowledge about which regions of noncoding DNA have regulatory (not just biochemical) function. Another major challenge is the inherent robustness of gene regulatory networks. At least within a laboratory context, sections of regulatory DNA can be removed with little apparent impact on phenotype or fitness (Ahituv et al. 2007). Similarly, divergent regulatory sequences from different species can be experimentally swapped with few detectable changes in gene expression across species (Borok et al. 2010). Developmental systems have built-in redundancy that can “buffer” the effects of regulatory mutations, for example, through compensation by other regulatory elements with partially overlapping activities (Hong et al. 2008; Frankel et al. 2010; Cannavò et al. 2016).

The complex relationship between DNA sequence and regulatory output further complicates our understanding of how mutations can impact gene regulation. For example, mutations affecting TF binding motifs can have a large impact on chromatin accessibility, Pol II occupancy, histone modifications, and gene expression (Kircher et al. 2019). But in some contexts/tissues, TF binding is driven by collective processes that can include protein–protein and protein–DNA interactions, such that mutations affecting a single TF motif may not substantially affect TF recruitment (Junion et al. 2012; Doitsidou et al. 2013; Uhl et al. 2016; Khoueiry et al. 2017). Moreover, many sequence variants affecting TF occupancy in vivo lie outside the TF's cognate motif and are likely owing to variation affecting the binding of co-occurring factors (Kasowski et al. 2010; Zheng et al. 2010; Reddy et al. 2012) or an overall change in DNA shape (Levo et al. 2015). To make matters more complex, enhancer output is not a strict function of all factors that occupy an enhancer; enhancers often contain binding sites for multiple factors with redundant input, and in some cases, different combinations of TFs can produce the same expression output (Brown et al. 2007; Zinzen et al. 2009; Khoueiry et al. 2017). Even in cases in which an enhancer's activity is abolished by mutations, gene expression may not be affected as genes often have many enhancers with partially overlapping activity (Hong et al. 2008; Frankel et al. 2010; Cannavò et al. 2016). With a few exceptions (Bullaughey 2011), this complex genotype-to-phenotype relationship cannot be modelled using regulatory sequence information alone but rather must be evaluated empirically (Khoueiry et al. 2017).

Allelic-specific data provide a unique opportunity to study the molecular mechanisms of *cis*-acting variation and have uncovered multiple regulatory processes through which *cis*-acting variation impacts transcriptional control (Kilpinen et al. 2013; Chen et al. 2016). F1 crosses of inbred strains provide an elegant method to determine the contribution of both *cis* and *trans* variation (Wittkopp et al. 2004; Tirosh et al. 2009; Goncalves et al. 2012; Wong et al. 2017). By using a multiline F1 design, we sought to better understand how natural sequence variation impacts different steps of gene regulation during embryonic development. We collected *Drosophila* F1 hybrid embryos from eight crosses and quantified allele-specific changes in TF occupancy (using open chromatin [ATAC-seq] as a proxy), enhancer and promoter activity (using H3K27ac or H3K4me3 and H3K27ac ChIP-seq as proxies, respectively), and gene expression (RNA-seq). By treating genetic variation affecting each of these regulatory layers as a perturbation to gene regulation, we could uncover functional relationships between different regulatory layers during embryonic development, as well as their impact on gene expression.

## Results

### Quantifying gene expression and regulatory element activity in hybrid embryos

**Figure 1.**
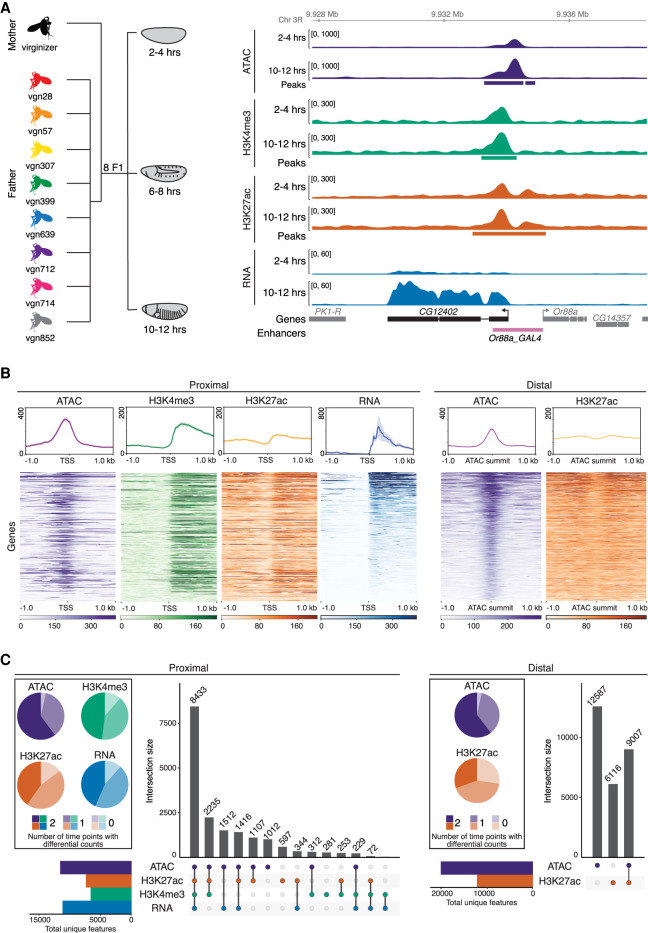
Quantifying gene expression and regulatory element activity in F1 embryos. (*A*, *left*) Experimental design and data structure. RNA-seq, ATAC-seq, and iChIP of H3K4me3 and H3K27ac were performed on embryos at three developmental stages from eight F1 hybrids with a common maternal line. (*Right*) Genome browser overview for the *CG12402* locus showing all data for 2–4 h and 10–12 h for the genotype vgn28. *Bottom* track shows curated enhancers (Kvon et al. 2014). (*B*, *top*) Density plots for representative cross (vgn × DGRP-639) at 6–8 h showing read count signal for TSS proximal and distal regions. Plots centered at TSS for promoter-proximal (*left*) and ATAC summits for distal (*right*) regions; shaded regions indicate 95% confidence intervals. (*Bottom*) Heatmaps showing quantitative signal of the same data as above, where rows were sorted by mean RNA-seq (proximal) or ATAC-seq (distal) signal. (*C*) *Upset* plots show colocalization of signal for proximal and distal regions for all four data types over all genotypes and stages. Regions common between data types (filled circle) are joined by a vertical bar. Horizontal bar plots indicate the number of unique genes/features. Pie charts indicate proportion of features with statistically different total read counts between time points (color indicates the number of times [0/1/2] the feature is differentially expressed).

To generate genetically diverse samples suitable for allele-specific analyses, we mated eight genetically distinct inbred lines from the *Drosophila melanogaster* Genetic Reference Panel (DGRP) collection (Mackay et al. 2012) to females from a common isogenic maternal line (Fig. 1A). The resulting half-sibling F1 panel contains an average of 567,412 SNPs per cross and a total of 1,455,988 unique SNPs covering a range of minor allele-frequencies and levels of conservation (phyloP scores) (Supplemental Fig. S1A; Supplemental Table S1).

Embryos were collected at three stages of embryogenesis: 2–4 h after egg laying, consisting primarily of pregastrulation, unspecified embryos (mainly stage 5); 6–8 h (mainly stage 11), when major lineages within the three germ-layers are specified; and 10–12 h (mainly stage 13), during terminal differentiation of tissues (Fig. 1A). For each developmental stage and F1 cross, we performed RNA-seq (gene expression), ATAC-seq (open chromatin), and iChIP for H3K27ac (marking active enhancers and promoters) and H3K4me3 (active promoters) (Buenrostro et al. 2013; Lara-Astiaso et al. 2014) from the same collection of embryos (four measurements × three stages × eight genotypes = 96 samples). In addition, we collected samples from the parental lines of one cross, forming a parent/offspring trio that allowed us to partition genetic differences between the parents into *cis* and *trans* (Wittkopp et al. 2004), defined here as genetic variation that affects the linked alleles of features on the same chromosome (*cis*) versus variation that affects both alleles on any chromosome (*trans*). All measurements were made in replicates, giving a total of 240 samples (192 F1 samples [96 × two replicates] + 48 parental [four measurement × three stages × two genotypes × two replicates]). Read counts were highly correlated between biological replicates, with median correlation coefficients of 0.98 for RNA, ATAC, and histone data (Methods) (Supplemental Fig. S1B). As expected, correlations were reduced between corresponding samples across genotypes, reflecting the functional impact of genetic variation (Supplemental Fig. S1C), and were reduced even further across time points, reflecting dynamic changes in gene expression during development.

To define noncoding features, ATAC-seq and ChIP-seq reads from each cross were mapped to each parental genome independently and the significant peaks merged into a combined peak set used for all subsequent analyses. In total, we identified 11,211 genes with detectable expression, 31,963 ATAC peaks, 19,769 H3K27ac peaks, and 6648 H3K4me3 peaks active at one or more stages (Supplemental Table S2). Of these, 93.9%, 95.8%, 95.2%, and 96.9%, respectively, contained at least one SNP that distinguishes maternal and paternal haplotypes in at least one line. The *CG12402* locus, a predicted ubiquitin-protein transferase, illustrates the dynamics of the data, transitioning from low to high expression from 2–4 h to 10–12 h (Fig. 1A), accompanied by quantitative changes in chromatin accessibility and, to a lesser extent, histone modifications in its promoter region.

To examine the regulatory relationships between these different signals, we divided noncoding features into promoter-proximal (<±500 bp of an annotated TSS or H3K4me3 peak [to capture unannotated promoters]) or -distal (putative enhancer; >±500 bp from a TSS) elements. At promoter-proximal regions, all signals show the expected enrichment and distribution around the TSS (Fig. 1B, proximal), showing the quality of the data. The ATAC-seq signal is highest directly at the promoter, representing occupancy of the basal transcriptional machinery, whereas H3K27ac and H3K4me3 signals are highest at the +1 nucleosome, reflecting the predominantly unidirectional nature of *Drosophila* promoters (Core et al. 2012; Mikhaylichenko et al. 2018). Although all three regulatory signals (ATAC-seq, H3K27ac, and H3K4me3) are highly correlated at promoters of actively transcribed genes (i.e., with RNA-seq signal), many promoter-proximal peaks (3907) marked by H3K4me3 with ATAC signal and/or H3K27ac show no detectable RNA signal (Fig. 1C, left upset plot). Many of these regions (approximately 850) correspond to known noncoding RNAs not captured by poly(A)^+^ RNA-seq libraries, suggesting the presence of many additional unannotated ncRNAs.

The majority of H3K27ac (62.8%) and ATAC peaks (63.9%) are distal to an annotated promoter. Distal ATAC peaks lacking H3K27ac signal (58% of the total) have a strong enrichment for Polycomb and Su(Hw) ChIP signal (Supplemental Table S3), suggesting that they represent repressed enhancers or other types of regulatory elements (e.g., insulators). For the remaining 9007 distal elements, H3K27ac signal is bimodally distributed around the ATAC-seq peak (Fig. 1B), suggestive of active enhancers. Although most H3K27ac peaks (60%) overlap ATAC peaks, many do not (Fig. 1C, right). This latter set often have ATAC signal below our threshold for peak calling (Supplemental Fig. S1D) and are enriched in a range of factors including Elav (Oktaba et al. 2015) and H3K27me3, suggesting that they represent a mixture of regulatory features, including enhancers with active and repressed states.

To quantify dynamic changes in each of the four regulatory layers across embryonic development, we pooled all F1 samples within a time point to formally test for changes in read counts across time (treating the data as 16 replicates per time point). Both proximal and distal sites, gene expression (RNA-seq), and noncoding elements (based on ATAC-seq and chromatin signatures) show similar dynamics, with the majority (72%–96%) of features showing statistically significant changes in total counts between developmental time points across all *F1* lines (Methods) (Fig. 1C, pie charts).

Taken together, these features show both the quality and richness of the data and their usefulness to further annotate the regulatory landscape of the *Drosophila* genome at these important stages of embryogenesis.

### Allele-specific variation is common across genotypes and regulatory layers

**Figure 2.**
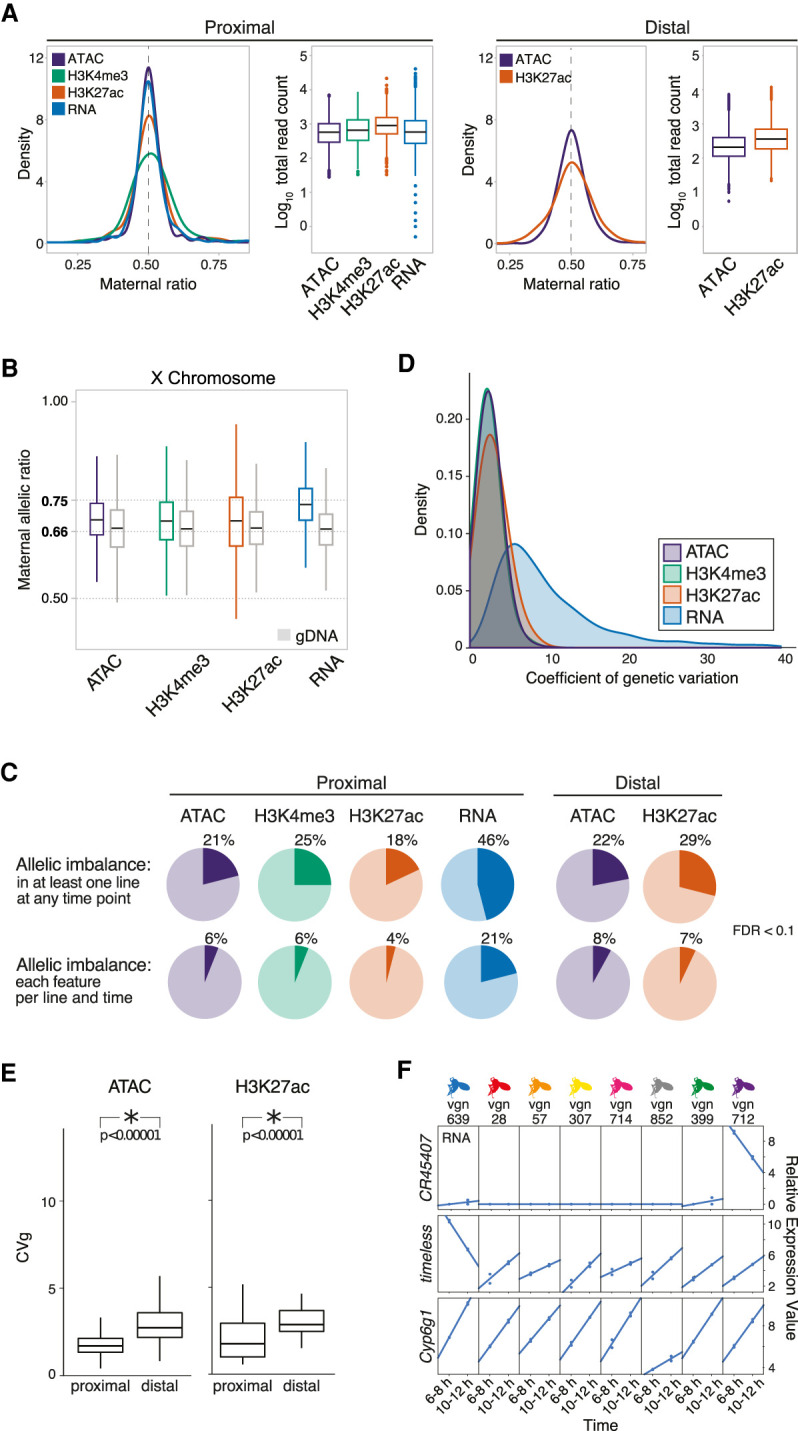
Allelic imbalance (AI) is common across regulatory data types. (*A*) Density plot of allelic count distribution for representative cross (vgn × DGRP-028) at 10–12 h and matching box plots showing total read count abundance (log_10_) in autosomes at TSS proximal (*left*) and distal (*right*) regions. Density plots for all crosses shown in Supplemental Figure S2B. (*B*) Box plot shows the distribution of maternal allelic ratios on the X Chromosome compared with genomic DNA (gray). (*C*) Pie charts show AI genes/features at promoter-proximal (*left*; TSS ±500 bp) and promoter-distal (*right*; 500–1500 bp ± from TSS) regions for all data types (FDR < 0.1). (*Top*) AI events in at least one F1 line at any time point. (*Bottom*) AI events detected in all eight F1 lines in all time points, on a per line and time basis. (*D*) Smoothed histograms showing distribution of coefficients of genetic variation for all features with significant between-line variances within each regulatory layer. (*E*) Box plots show the distribution of coefficient of genetic variation (CVg; *y*-axis) for chromatin accessibility (*left*) and H3K27ac signal (*right*), for promoter-proximal and promoter-distal sites. Genetic influences are more pronounced at distal elements in ATAC and H3K27ac. (*F*) Three examples of individual lines having distinct expression variation on specific genes. Relative expression values are typically larger for RNA than noncoding features, an effect that often results from one or two lines as shown here.

To quantify the impact of *cis*-acting genetic variation, we compared the number of reads mapping to the maternal and paternal chromosomes in each F1 cross, using informative SNPs to assign reads to parent of origin (Methods) and an empirical Bayes framework to formally test for imbalance in the ratio of maternal:paternal reads at each locus per line and time point combination (Supplemental Fig. S2A). As expected, most genes and regulatory features had allelic ratios centered at 50:50 across autosomes (Fig. 2A; Supplemental Fig. S2B), with a slight elevation in the magnitude of allelic imbalance (AI) at distal sites (Supplemental Fig. S3A). RNA allelic ratios were also concordant with the direction of change of embryonic eQTL (Supplemental Fig. S3B), previously quantified in the same paternal lines at the same stages of embryogenesis (Cannavò et al. 2017), further verifying our approach and the quality of the data. The early embryonic time point (2–4 h) is an expected exception to this balanced maternal/paternal ratio owing to maternally deposited transcripts and the presence of unfertilized eggs (Supplemental Fig. S2B). We therefore removed the 2–4 h time point RNA samples from all allele-specific analyses.

To evaluate sex ratios in the embryo pools and to set a reference point for evaluating AI and dosage compensation on the X Chromosome (Lucchesi and Kuroda 2015), we sequenced the genomic DNA (gDNA) of each cross. This confirmed that our embryonic pools were relatively sex balanced, with the expected X Chromosome allelic ratio of approximately 0.66 observed for gDNA (Fig. 2B). Sex-chromosome dosage compensation in *Drosophila* is achieved by a two-fold up-regulation in gene expression of X Chromosome genes in XY males (Georgiev et al. 2011). Consistent with this, we observed a maternal:paternal ratio of 0.74 for RNA, closely matching the expected 0.75 ratio (Methods) (Fig. 2B). A proposed hypothesis for this twofold up-regulation in gene expression on the male X Chromosome is that it is due to a twofold increase in the loading of polymerase at the corresponding promoter (Conrad et al. 2012). However, the maternal:paternal ratio that we observe for chromatin data does not fully reflect this up-regulation: For both chromatin accessibility and histone modifications, the observed ratios at X Chromosome sites are more similar to the observed genomic ratio of 0.66 than to the expected 0.75 ratio under full dosage compensation (H3K27ac = 0.688, H3K4me3 = 0.692) (Fig. 2B). This indicates that dosage compensation in *Drosophila* does not involve a linear increase in chromatin accessibility on the male X Chromosome (although we do observe a slight increase in accessibility; ATAC = 0.693) (Fig. 2B; Urban et al. 2017; Pal et al. 2019). Regardless of its cause, we used the empirically observed average ratio for X Chromosome features for each data type to form the null hypothesis in subsequent beta-binomial tests for AI.

Overall, AI is common, with 46% of genes and between 18% and 25% of noncoding chromatin features showing statistically significant AI in at least one line at one or more time point (FDR < 0.1) (Fig. 2C). The magnitude of AI is generally evenly distributed across SNPs with a range of minor allelic frequencies. However, highly imbalanced peaks show a strong enrichment for extremely rare SNPs found uniquely in the maternal line relative to the DGRP panel (χ^2^ test, *P* < 2.2 × 10^−16^) (Supplemental Fig. S3C), highlighting the disproportionate impact of rare mutations on expression phenotypes (Cannavò et al. 2017).

AI is more frequently observed for RNA than for other regulatory layers (Fig. 2C), in contrast to mammals (Goncalves et al. 2012; Wong et al. 2017). Additionally, *Drosophila* promoter-proximal sequences appear to evolve more rapidly than distal elements (putative enhancers); proximal elements are slightly more polymorphic (pair-wise differences [pi] = 0.132 vs. 0.129; Wilcoxon test, *P* = 1 × 10^−10^) and evolve faster (phyloP = 0.514 vs. 0.560; Wilcoxon test, *P* < 2.2 × 10^−16^) (Supplemental Table S4). Despite this, distal peaks of open chromatin and H3K27ac show larger (Tukey's ASD, *P* < 1 × 10^−4^) and slightly more frequent (χ^2^ test, *P* < 2.2 × 10^−16^) AI than their proximal counterparts (Supplemental Fig. S3A).

To understand how allelic imbalance relates to heritable variation at the total count level, we took advantage of the fact that our measured F1 lines share a common maternal line but have unrelated, genetically diverse paternal lines. As a result, differences among F1s are expected to be proportional to heritability, or the degree to which phenotypic variation can be explained by genetic factors (Lynch and Walsh 1998). Expressed as percentage deviation from the mean phenotype (coefficient of genetic variation), the impact of genetic variation on chromatin features is relatively modest, with the average peak varying by ∼5%–10% of the mean phenotype among crosses (Fig. 2D). The magnitude of heritability genetic variation is generally higher at distal, compared with proximal, regulatory elements (*P* < 1 × 10^−5^; Methods) (Fig. 2E), consistent with the greater magnitude of AI at distal sites. In contrast to chromatin features, the magnitude of effects is generally higher for RNA, with an average coefficient of genetic variation of ∼9% and a tail extending to ∼40%. In many of these cases, high coefficients are driven by one or a few lines showing highly divergent patterns of expression (Fig. 2F), suggesting the presence of large effect, likely *cis*-acting mutations.

### AI is pronounced in metabolism and environmental response genes

Categorical enrichment for AI (Methods) identifies more extensive imbalance for genes associated with fast-evolving, *Drosophila*-specific genes (Mi et al. 2003; Turner et al. 2008) and metabolic genes, whereas TFs and their associated regulatory elements are depleted (Supplemental Fig. S4; Supplemental Table S5), consistent with our previous eQTL study (Cannavò et al. 2017). Selection may play a role in these AI differences; regulatory regions in the vicinity of TFs show reduced nucleotide diversity (pi, rank biserial correlation = −0.052; *P* < 1 × 10^−4^) and harbor more low-frequency SNPs (rank biserial correlation = −0.173; *P* = 2.8 × 10^−3^) (Supplemental Table S5) compared with background. However, this difference in AI may also be explained by different sensitivities between gene categories to mutations, a point we explore below.

For most gene categories, AI is equally likely to favor the maternal or the paternal allele. However, immunity and insecticide resistance genes show a clear paternal bias (Supplemental Fig. S5A; Supplemental Table S6). *Cyp6g1*, for example, is not expressed in embryos of our laboratory-derived maternal line (Supplemental Fig. S5B) but is strongly up-regulated in every measured paternal haplotype from the wild, and its expression contributes to DDT resistance in multiple *Drosophila* species (Supplemental Fig. S5B; Daborn et al. 2001; Battlay et al. 2016). Highly imbalanced genes like *Cyp6g1* (Supplemental Table S6) often overlap genes whose expression varies extensively among lines (*P* < 1 × 10^−6^) (Supplemental Fig. S5C) and have high levels of heritability, suggesting a close link between *cis-*acting variation and selection to changing environments.

### The impact of *cis*-acting genetic variation is largely consistent across development

Gene expression is highly dynamic across development (Arbeitman et al. 2002) and is largely driven by dynamic changes in enhancer usage (Wilczyński and Furlong 2010; Reddington et al. 2020). However, the extent to which this dynamism shapes the impact of regulatory variation is not well understood. We thus examined how allelic ratios at individual loci changed during embryogenesis.

**Figure 3.**
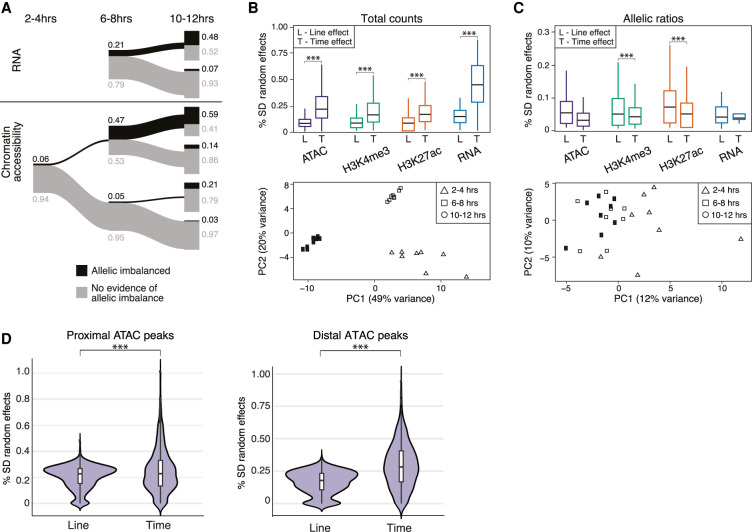
AI is generally not predictive of developmental time. (*A*) The relationship of AI across time for RNA (*top*) and chromatin accessibility (*bottom*). Proportions of AI and non-AI features shown in black and gray, respectively, and represented by the thickness of line (exact numbers indicated). Data for RNA at 2–4 h are not included owing to presence of maternal transcripts. (*B*, *top*) Box plots show distribution of time (T) and line (L) effect sizes obtained from mixed linear models for total counts. (*Bottom*) Principal component analysis (PCA) of gene expression for total counts. (*C*, *top*) Box plots showing the distribution of time (T) and line (L) effect sizes obtained from mixed linear models for allelic ratios. (*Bottom*) PCA of gene expression for allelic ratios. (*D*) Results from mixed linear models examining the effect of developmental time versus line (genotype) between proximal and distal ATAC-seq peaks for total count data. Distal peaks show a larger time effect compared with genotype effect (Mann–Whitney *U* test, *P* < 2.2 × 10^−61^). This is only slightly evident for promoter-proximal peaks (Mann–Whitney *U* test, *P* < 8.5 × 10^−5^).

Overall, we observed considerable constancy of AI between embryonic time points; imbalanced features at one time point have a ∼50% chance of being imbalanced in the subsequent time point (Fig. 3A; Supplemental Fig. S6A). To further quantify this, we constructed a series of linear models comparing the effect sizes of genetics (genotype/line) versus developmental stages (time), and the interaction between the two (GxT), for both total counts and allelic ratios.

For total counts, developmental time was the greatest contributor to variation across all data types (Fig. 3B, upper panel), consistent with the clear time-specific clustering by principal component analysis (PCA; Methods) (shown for RNA in Fig. 3B, lower panel). Time effects are more pronounced at distal compared with proximal ATAC peaks (Fig. 3D), reflecting both the constitutive accessibility of many promoters and the dynamic usage of enhancers during development (Wilczyński and Furlong 2010; Reddington et al. 2020). Interaction effects (GxT) occur frequently and are particularly common for gene expression, making up ∼30% of all analyzed models (Supplemental Table S7), consistent with our previous analyses at the total count level (Cannavò et al. 2017).

In contrast, the impact of time is significantly reduced compared with genetic effects for allelic ratios (Fig 3C, upper panel). Correspondingly, there is a lack of time point–specific clustering of allelic ratios in PCA (Fig 3C, lower panel), although there are some examples of allelic ratios that change over time in a coordinated manner between regulatory layers (Supplemental Fig. S6B). Unlike total counts, there is little evidence for interaction effects (Supplemental Table S7), consistent with the rarity of gene × environment effects reported for AI (Moyerbrailean et al. 2016; Knowles et al. 2017).

In summary, allelic effects are often larger at distal compared with promoter regions, with effects at both regions being largely consistent across developmental time points. In contrast, total counts vary markedly between embryonic time points, with interactions between genotype and developmental stage (GxT) being common.

### Genetic variation affects gene expression through chromatin by two different mechanisms

**Figure 4.**
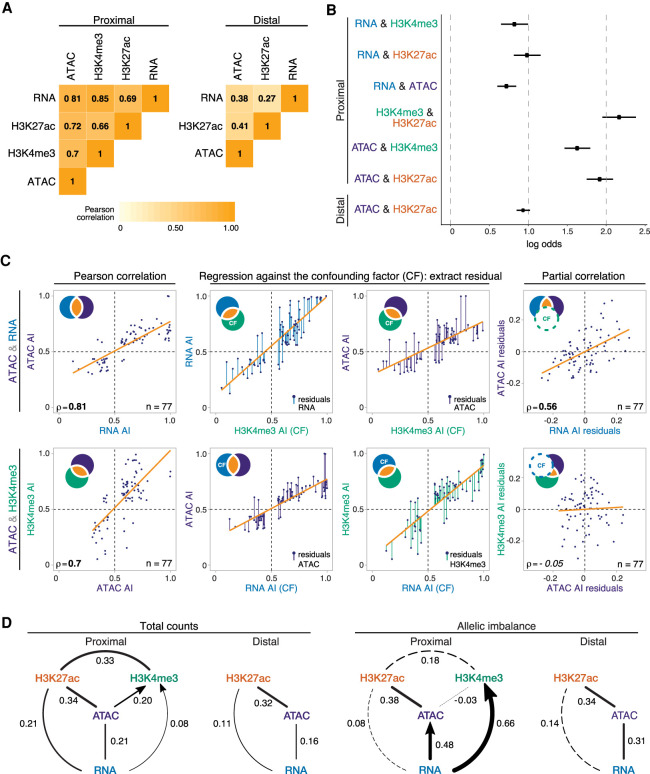
AI is propagated through regulatory layers via different epigenetic paths. (*A*) Heatmaps show Pearson correlation coefficient of allelic ratios between each data type for promoter-proximal (*left*) and promoter-distal (*right*) regions. Data restricted to 6–8 h and 10–12 h and features/genes whose allelic ratio exceeds 0.5 ± 0.06. (*B*) Log odds (intersection-union test) of co-occurrence of AI between two regulatory layers: 6- to 8-h and 10- to 12-h data shown; bars (from dots), 95% confidence intervals. (*C*) Stepwise example of partial correlation analysis of allelic ratios between chromatin accessibility and gene expression (ATAC&RNA; *top*), and promoter-proximal chromatin accessibility and H3K4me3 (ATAC&H3K4me3; *bottom*). Venn diagram schematics (*top left*) illustrate the variance of each variable and its shared proportion (orange), measured by linear regression (orange lines). (*Left* panels) Pearson's correlations for the two comparisons are significant. (*Middle*) Regression of each initial variable against a third, confounding variable (H3K4me3, *upper* row; RNA, *lower* row). Residuals of the initial variables (colored lines) represent the nonoverlapping part of the circle of the same color in the schematic. (*Right*) Correlations of the residuals, excluding shared variance by the confounding factor (dashed circle in schematic). This resulting partial correlation is significant in the *top*, and not in the *bottom*, examples. (*D*) Partial correlation and directional dependency regression for total counts (*left*) and allelic ratios (*right*). Significant partial correlations (solid lines) suggest direct dependencies among regulatory layers. For each significant edge (*P* < 0.01), copula regression was used to assign directionality (arrows; delta > 0.01). Line thickness indicates the value of partial correlations; dashed lines indicate nonsignificance. Promoter-proximal and promoter-distal results shown separately.

Although highly correlated, the causal relationships between chromatin accessibility, histone modifications, and gene expression remain unclear. To assess this, we used allelic ratios as a perturbation to different regulatory layers and modelled the paths by which genetic variation impacts regulatory phenotypes. Like total counts, allelic ratios are highly correlated among regulatory layers (Fig. 4A; Supplemental Fig. S7), and in all cases, we could reject the null hypothesis of independence (all *P*-values < 4.2 × 10^−17^). Co-occurrence of statistically significant imbalance (intersection-union test, FDR < 0.1) is pronounced for chromatin features, in particular H3K4me3 and H3K27ac, at promoter regions, with a log-odds greater than 2.0 (Methods) (Fig. 4B). For chromatin accessibility and H3K27ac, the co-occurrence of AI is more pronounced at promoters than enhancers (distal) (Fig. 4B), despite AI being more frequent (*P* < 2.2 × 10^−16^) (Fig. 2C) and being of greater magnitude (Supplemental Fig. S3A) at distal sites. This suggests that H3K27ac and chromatin accessibility are more functionally coupled at promoters compared with enhancers, perhaps reflecting the fact that not all active enhancers seem to require H3K27ac (Bonn et al. 2012; Pradeepa et al. 2016).

To identify potentially causal relationships across regulatory layers, we used partial correlation to identify independent, pairwise correlations between multiple covarying variables beyond their global correlations, after thresholding on allelic ratios to remove features/genes with low information content (Methods) (Fig. 4C; Supplemental Fig. S8A; Lasserre et al. 2013; Pai et al. 2015). For total count data, our results closely mirror those of Lasserre et al. (2013) in CD4^+^ and IMR-90 cells, including finding a clear relationship between gene expression levels and the total abundance of H3K27ac that is independent, at least at a statistical level, of the correlation between expression and H3K4me3 (i.e., there is little independent correlation between changes in RNA and changes in H3K4me3 total counts) (Fig. 4D, left). We also observed a statistically significant relationship between open chromatin and gene expression, confirming that although RNA levels are influenced by post-transcriptional processes, genetic variation at *cis*-regulatory elements contributes directly to differences in gene expression among individuals.

This relationship is even more pronounced for allelic ratios, which show a clear link between open chromatin and gene expression at both proximal and distal elements (Fig. 4D, right). H3K27ac and open chromatin are also significantly correlated at promoters, although we see little evidence for a direct relationship between H3K27ac and gene expression itself (Fig. 4D, right). The latter is a marked difference from what is observed with total count data and suggests that although H3K27ac at promoters is highly correlated with, and even predictive of (Karlic et al. 2010), gene expression, they may not be mechanistically directly linked. In contrast, allelic ratios for promoter-proximal H3K4me3 show strong evidence of a direct correlation with gene expression that is independent of allelic differences in chromatin accessibility or H3K27ac (Fig. 4D, right). Taken together, this analysis suggests two independent pathways by which segregating mutations influence gene expression: one affecting open chromatin and promoter-proximal H3K27ac and the other influencing H3K4me3.

To explore these relationships further, we analyzed each edge identified by partial correlations using copula directional dependence analysis (Kim et al. 2008; Lee and Kim 2019), a statistical approach based on copula regression that evaluates the directionality of pairwise relationships while allowing for nonlinearities (Methods). For TSS-proximal regions, our analysis placed RNA upstream of both H3K4me3 and open chromatin (Fig. 4D, right, arrow). Although counterintuitive at first glance, this suggests that gene expression is relatively robust to variation in H3K4me3, whereas conversely, variation in RNA is more predictive of H3K4me3 signal. This may reflect buffering processes but is also consistent with the hypothesis that H3K4me3 is not functionally required for transcription but is rather deposited as a consequence and may be involved in post-transcriptional events (Howe et al. 2017). Similarly, allele-specific variation in RNA better explains variation in chromatin accessibility compared with the reverse; that is, not all variation in open chromatin leads to a corresponding change in gene expression (Fig. 4D, right).

In summary, by measuring informative dependencies on the impact of *cis*-acting genetic variation, we identified multiple epigenetic pathways affecting transcription. Specifically, genetic variation acts to change gene expression levels via the interplay between at least two different promoter-proximal paths: open chromatin and H3K27ac, or H3K4me3. Moreover, the flow of information suggests that gene expression is often buffered against *cis*-acting mutations (presumably affecting TF binding) at associated regulatory elements, although we cannot rule out post-transcriptional processes that may also help to buffer allelic ratios.

### Regulatory buffering varies depending on gene function and local chromatin architecture

**Figure 5.**
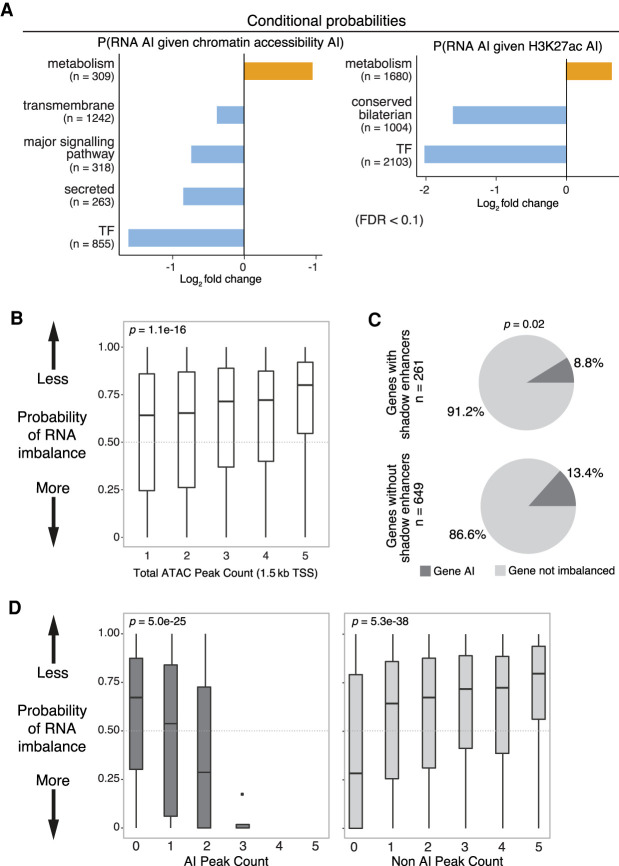
Regulatory buffering varies across gene categories and with local chromatin structure. (*A*) Conditional probability of AI in gene expression given AI in associated chromatin (*left*) and H3K27ac (*right*) peaks across gene categories. *X*-axis shows log_2_ fold change; background is genome-wide expectation. Gene categories enriched (orange) or depleted (blue) for imbalance are indicated (FDR > 0.1, Fisher's exact test). n = number of genes per category combined across all lines and both 6- to 8-h and 10- to 12-h samples. (*B*) Box plots show probabilities of AI in gene expression based on numbers of neighboring ATAC peaks (TSS < 1.5 kb). Genes with more ATAC peaks are less likely to show AI. (*C*) Pie charts show the proportion of genes with AI in RNA associated to ATAC-seq peaks overlapping known partially redundant/shadow enhancers (*top*) or not (*bottom*). Genes associated with shadow enhancers are less likely to have AI (determined using a beta-binomial model; *x*^2^=5.3, *P* = 0.02). (*D*) Box plots show the probability of AI in gene expression (*P*-value from a beta-binomial model; *y*-axis) as a function of the number of ATAC-seq peaks with AI (*left*) or not imbalanced (*right*).

Genes often differ in the complexity of their regulatory landscapes. Metabolic genes, for example, typically have relatively simple and compact regulatory landscapes with few enhancers that are located close to the gene's promoter (Zabidi et al. 2015; Corrales et al. 2017). TFs, in contrast, have many enhancers often with partially overlapping spatial activity (“shadow enhancers”), located at varying distances from the gene's promoter (Spitz and Furlong 2012; Long et al. 2016), which may help to buffer TFs against mutations in regulatory DNA (Xiong et al. 2002; Cretekos et al. 2008; Montavon et al. 2011; Cannavò et al. 2016; Lu and Rogan 2018). We evaluated this hypothesis in two ways. First, we assessed the extent to which AI in the expression of different gene categories is independent of, or buffered from, imbalance in their associated regulatory elements using conditional probabilities. Among all comparisons, the expression of ancient genes (conserved bilaterian processes) and of genes coding for TFs, transmembrane proteins, and signaling components is most robust to imbalance in their regulatory regions, whereas genes involved in metabolism have high sensitivity (Fig. 5A, cf. blue and orange).

Second, we directly assessed the relationship between AI and regulatory complexity (ATAC-seq peak number within a gene's ± 1.5-kb TSS regulatory domain). Imbalanced genes have fewer associated ATAC peaks genome-wide (Kruskal–Wallis, *P* = 1.1 × 10^−16^) (Fig. 5B), a trend that is most pronounced for single-peak genes, which have significantly more AI than genes associated with multiple regulatory elements (Mann–Whitney *U* test, *P* = 6.4 × 10^−6^). Furthermore, genes associated with previously characterized, partially redundant “shadow enhancers” (Cannavò et al. 2016) have a modest reduction in the frequency of AI compared with genes without (*x*^2^ = 5.3, *P* = 0.02) (Fig. 5C). However, AI at multiple enhancers in the vicinity of a gene can have a cumulative influence on gene expression, as genes with multiple regulatory elements are more likely to be imbalanced when multiple associated peaks show unbalanced allelic ratios (Fig. 5D).

In summary, the degree to which a gene's expression is influenced by noncoding genetic variation in its regulatory elements is influenced by the gene's regulatory complexity, with more regulatory elements providing a degree of buffering against genetic perturbations.

### *Trans*-acting variation influences the heritability of gene expression

Regulatory differences between individuals may also be influenced by *trans*-acting genetic variation. To assess the influence of *trans*-acting variation, we measured the same regulatory features in embryos from our maternal line (vgn) and one paternal line (DGRP-399), forming a trio of embryos from both parental lines (F0) and their F1 embryos. This allowed us to quantify *trans* effects by comparing the difference between parental lines (from both *cis*- and *trans*-acting variants) to allelic ratios (*cis* only) measured in the F1 (Landry et al. 2005; Tirosh et al. 2009; Goncalves et al. 2012; Wong et al. 2017) using a maximum likelihood framework that classified genes and features as *cis*, *trans*, *cistrans*, or *conserved* (Wong et al. 2017).

**Figure 6.**
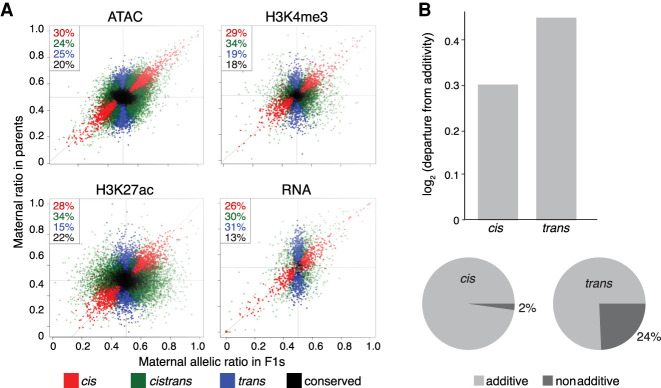
Chromatin features are more heritable than gene expression. (*A*) Scatter plots of F1 allelic ratio (*x*-axis) against the maternal/paternal ratio observed in (normalized) parental, total count libraries (*y*-axis). Genes/features along the diagonal are exclusively influenced by *cis*-acting variation, whereas vertically distributed genes/features show exclusively *trans* influences. Colors indicate maximum likelihood classification into *cis*, *trans*, and *cistrans* (a mixture of *cis* and *trans*) or conserved genes/features. (*B*, *top*) Bar plots showing the magnitude of deviation from additivity (parental mean) for *cis* versus *trans* (BIC ≥ 2) features. (*Bottom*) Pie charts showing fraction of additive and nonadditive genes for *cis* (*left*) and *trans* (*right*) classes.

Among noncoding chromatin features, *cis*-acting effects are more common than *trans* (59% vs. 41%; *P* < 2.2 × 10^−16^, χ^2^; Methods) (Fig. 6A; Supplemental Table S8). This enrichment is particularly pronounced for histone modifications, with nearly twice as many *cis* influenced peaks compared to *trans* (Fig. 6A; Supplemental Fig. S9A). For both open chromatin and H3K27ac, *cis* effects are slightly more common at promoters than enhancers (ATAC = 0.32 vs. 0.29, H2K27ac = 0.30 vs. 0.27; *P* < 1 × 10^−4^).

Gene expression, in contrast, is more strongly influenced by *trans*-acting genetic variation (55% *trans* vs. 45% *cis*; *P* = 0.0073, χ^2^) (Fig. 6A). Moreover, a higher fraction of *cistrans* genes have more *trans*, compared with *cis*, variation (*trans* proportions 0.67 vs. 0.53; *P* = 2.77 × 10^−5^) (Supplemental Fig. S9A), whereas *cis* and *trans* are balanced for *cistrans* classified noncoding features.

Previous studies suggest that *trans*-influenced features generally show nonadditive inheritance (Lemos et al. 2008; Meiklejohn et al. 2014; Wong et al. 2017) and are thus less likely to be directly influenced by natural selection (Lynch and Walsh 1998). Our data suggest that open chromatin features, whether influenced by *cis* or *trans*, have primarily additive inheritance (Supplemental Fig. S9B), consistent with the finding that most variation affecting TF binding is inherited additively (Wong et al. 2017). In contrast, for gene expression, an additive model could be rejected for 32% of genes, with *trans* influenced genes departing from an additive model more frequently than *cis* (24% vs. 2%; χ^2^, *P* < 1 × 10^−4^) (Fig. 6B). *Trans* effects are most common for genes associated with complex regulatory landscapes (Supplemental Table S9), and correspondingly, genes showing nonadditive inheritance have more ATAC peaks (2.19 peaks per gene vs. 1.82; Wilcoxon test, *P* = 1.4 × 10^−3^). This suggests that in addition to buffering genes from *cis*-regulatory variation, complex regulatory landscapes can influence patterns of heritability, with downstream consequences for how selection can act on gene expression phenotypes (Supplemental Fig. S9).

## Discussion

In this study, we generated an extensive F1 data set to better understand the functional impact of genetic variation in regulatory DNA on embryonic gene expression and to shed light on how these effects are propagated or buffered through different regulatory layers. Our analysis revealed several new insights into the impact of regulatory mutations on transcriptional phenotypes.

First, although *cis*-acting genetic variation is common in development, its effects are not equally distributed across the genome. Allelic variation both is more frequent and has greater magnitude at distal regulatory elements (putative enhancers) compared with promoters, despite genetic variation itself being more common at promoters. This may in part be owing to differences in the relative importance of sequence content at promoters and enhancers: Many promoters, particularly for broadly expressed genes, have high tolerance to genetic variation (Schor et al. 2017). But despite having a greater magnitude, AI at distal elements is less likely to be propagated to other regulatory layers (Fig. 3), suggesting that enhancer mutations are often effectively buffered, a hypothesis that fits well with the observed robustness of gene expression to deletions that remove distal regulatory elements (Hong et al. 2008; Cannavò et al. 2016).

Second, although all data types (open chromatin, histone modifications, RNA levels) are highly correlated, their explanatory values (potential causal relationships) as revealed by partial correlation analysis are not equal. By using *cis*-acting variation as perturbations to development, we observed a strong, likely direct, relationship between variants affecting open chromatin (TF binding) at proximal and distal sites to a degree that was not observed at the total count level. We also uncovered a strong, potentially causal, link between allelic imbalance in H3K4me3 signal and AI in the expression of the corresponding genes, which was largely independent of imbalance in H3K27ac signal. Our copula analysis placed H3K4me3 downstream from RNA (Fig. 4D), suggesting that although AI at the RNA level is predictive of AI in H3K4me3, mutations impacting H3K4me3 often do not directly impact RNA. This placement of RNA upstream of H3K4me3, inferred from our analysis of the functional impact of genetic variation, is supported by recent genetic ablation studies showing that RNA transcription does not require H3K4me3 (Clouaire et al. 2012, 2014; Margaritis et al. 2012) and is consistent with suggestions that H3K4me3 is deposited as a consequence of transcription and may be required in more downstream post-transcriptional events (Howe et al. 2017).

Third, the impact of *cis*-acting variation on gene expression is influenced by regulatory complexity, with genes having more regulatory elements being less likely to show AI (Fig. 5). This may reflect selection against variation in regulatory elements associated with these genes, and indeed, we observe less AI in regulatory elements associated with developmental regulators, extending previous findings (Cannavò et al. 2016). But even accounting for reduced overall AI, TFs and other genes with complex regulation show more independence from AI in associated regulatory layers. Although we cannot rule out a role for allele-specific post-transcriptional processes (Pai et al. 2012; Sun et al. 2018), these results suggest an active buffering process resulting from the presence of multiple regulatory inputs (Lu and Rogan 2018; Waymack et al. 2020). That said, as the number of regulatory elements with AI near a gene increases, so does the probability that the gene will show AI, suggesting that such buffering is not absolute.

Finally, *trans*-acting variation is more common for RNA than other regulatory layers, particularly for genes with complex regulatory landscapes such as TFs. This later observation, likely owing to the buffering effects of complex *cis* regulatory landscapes (Scholes et al. 2019; Waymack et al. 2020), has potentially counterintuitive evolutionary consequences: Although reduced in genetic variation overall, predominantly *trans*-influenced genes are more likely to show nonadditive, and thus less selectable, patterns of inheritance. As a result, *trans*-acting variation in genes such as TFs may remain in populations even as negative selection and buffering act to reduce the influence of *cis*-acting mutations. Why *trans*-acting variation is more common for RNA is not immediately clear but may reflect the accumulation of variation impacting both transcriptional and post-transcriptional processes (Liu et al. 2019).

In summary, allelic variation in chromatin accessibility and histone modifications at regulatory elements is prevalent and capable of propagating across regulatory layers. The extent of this information flow, or propagation, depends on the type of regulatory element and appears mitigated at developmental regulators.

## Methods

### Fly husbandry, crosses, and embryo collection

F1 hybrid embryos were generated by crossing males from eight genetically distinct inbred lines from the DGRP collection (Mackay et al. 2012) to females from a common maternal “virginizer” line. The virginizer line contains a heat-shock-inducible proapoptotic gene (*hid*) on the Y Chromosome (Starz-Gaiano et al. 2001) of a laboratory reference strain (*w*^*1118*^) that kills all male embryos after a 37°C heat-shock.

### RNA-seq, ATAC-seq, and iChIP

For three developmental stages (2–4 h, 6–8 h, and 10–12 h after egg laying), we performed RNA-seq, ATAC-seq, and iChIP for H3K27ac and H3K4me3 for pooled embryos of each F1 strain. All experiments were performed in biological replicates from independent embryo collections. iChIP experiments were performed as previously described (Lara-Astiaso et al. 2014). Detailed library information can be found in the Supplemental Methods, pages 13 through 15.

### Sequencing read processing

Strain-specific genomes and annotations were constructed by inserting genetic variants and indels into the *Drosophila* dm3 assembly (version 5 from FlyBase) and translating the reference annotations (r5.57) to the personalized genomes using pslMap (Zhu et al. 2007). ATAC-seq and ChIP-seq reads were mapped using BWA (Li and Durbin 2010), whereas RNA-seq reads were mapped using STAR (Dobin et al. 2013) followed by quality filtering and the assignment of reads to parent of origin using SNP overlaps (for more details, see page 16 in the Supplemental Methods).

### Demarcation of distal peaks

We evaluated the Spearman's correlations of allelic ratio between ATAC-seq and histone mark peaks to gene expression based on peaks at increasing distances from the genes’ TSS up to a distance of 10 kb. We defined a distance of 1.5 kb from the TSS to be a conservative demarcation between proximal and distal peaks based on the presence of reasonable correlations between genes and associated peaks at this distance.

### Test for allele-specific imbalance

Because of the extensive maternally deposited transcripts still present at 2–4 h, we excluded the RNA-seq data from this time point from all downstream allele-specific analysis to avoid potential confounding effects in AI measurements. To test for AI, an empirical Bayesian statistical framework was used to test the null hypothesis for differences in read counts between F1 alleles for each feature of each data set (RNA-seq, ATAC-seq, H3K4me3, H3K27ac) (see page 21 of the Supplemental Methods).

### Allele-specific changes across lines and developmental time

A linear mixed-effects model, in which random effect components were incorporated, was used to estimate variability between pools of individuals, time points and lines:

$$y_{f}^{d,s,r,t}=\;\mu_{f}+\delta_{f}^{t}+\omega_{f}^{s}+{\left(\delta \omega \right)}_{f}^{t}\;\omega_{f}^{s}∼N(0,\sigma_{f}^{2})$$

*μ*_*f*_ is the intercept term, $\delta_{f}^{t}$ is a random effect term denoting time, $\omega_{f}^{s}$ is a random effect based on strain, and ${\left(\delta \omega \right)}_{f}^{t}$ is a interaction term for time by strain.

To infer the significance of time- or strain-dependent allele bias, we restricted the values that the parameters can take. Library size differences were corrected for at the allele-combined count level using the TMM method in “edgeR” (Robinson et al. 2010) before analysis. Not all features contained enough information for statistical testing; analyses were limited to features with at least six samples in each of the three time points in at least four genetic strains.

### Allele-specific changes across regulatory layers

Intersection-union tests were used to examine the pairwise co-occurrence of AI in overlapping genes/features, limited to autosomes, based on rejecting the null hypothesis if a significant outcome with respect to the feature compared at the same time point exists for both data types (Berger and Jason 1996).

To infer pairwise relationships between regulatory data types while reducing indirect relations, partial correlation analysis was performed using “GeneNet” (Opgen-Rhein and Strimmer 2007) for both allelic ratios and total count data. Directional dependence modeling was performed in a regression framework using copulas (Lee and Kim 2019) to infer the flow of information for significant pairwise relationships in partial correlation analyses (see page 26 of the Supplemental Methods).

Conditional probabilities for the probability of AI given imbalance in a different regulatory data type were calculated by the following definition:

$$P(A|B)=\;\frac{P(A\cap B)}{P(B)},$$

where *A* and *B* are the probabilities of AI in each data type.

### *Cis/trans* analysis

For one F1 line (vgn × 399) and its parental lines, maximum likelihood estimation (MLE) was used to compare parental and offspring ratios simultaneously to determine whether gene expression, chromatin accessibility, or H3K4me3 and H3K27ac enrichments are influenced by *cis*-acting, *trans*-acting, conserved, or *cistrans*-acting effects by modeling read counts. For parents, the total count data were modeled using negative binomial distributions whereas allelic differences in F1s were modeled using beta-binomial distributions (Supplemental Methods). We constrained parameter estimation for each model based on four different regulatory scenarios and derived maximum likelihood values for each feature (shown in Fig. 6A; Supplemental Fig. S9C). In subsequent analyses, we limited analyses to features that showed a BIC difference equal or greater than two.

### Measuring additive versus nonadditive heritability

Additive inheritance implies that the F1 signal is equal to the midpoint (average) of the two parents. Nonadditive inheritance in this analysis was thus determined by testing for departure of the F1 from the parental midpoint using DESeq2 (Love et al. 2014).

## Data access

All raw data generated in this study have been submitted to the EMBL-EBI ArrayExpress database (https://www.ebi.ac.uk/arrayexpress/) under accession numbers E-MTAB-8877 (gDNA), E-MTAB-8878 (RNA-seq), E-MTAB-8879 (ATAC-seq), and E-MTAB-8880 (ChIP-seq H3K4me3, H3K27ac). Processed data, including total counts, allelic ratios, *cis*/*trans* estimates, estimated per-feature heritability, mappability filters, and parental genotype files, can all be downloaded from http://furlonglab.embl.de/data.

## Competing interest statement

The authors declare no competing interests.

## Supplementary Material

Supplemental Material
