# Supplementary material for: Cis-acting variation is common across regulatory layers but is often buffered during embryonic development
Source: Genome Res. 2021 Feb;31(2):211–24. doi: 10.1101/gr.266338.120 (PMC7849415; doi:10.1101/gr.266338.120)
Supplement: Supplemental Material [file supp_31_2_211__index.html]

Cis-acting variation is common across regulatory layers but is often buffered during embryonic development — Cis-acting variation is common across regulatory layers but is often buffered during embryonic development — Supplemental Material 

# *Cis*-acting variation is common across regulatory layers but is often buffered during embryonic development

## Supplemental Material

- SupplementalTables.zip
- Supplemental\_Figures\_and\_Methods.pdf
