# Supplementary material for: Cis-acting variation is common across regulatory layers but is often buffered during embryonic development
Source: Genome Res. 2021 Feb;31(2):211–24. doi: 10.1101/gr.266338.120 (PMC7849415; doi:10.1101/gr.266338.120)
Supplement: Supplemental Material [file supp_gr.266338.120_Supplemental_Figures_and_Methods.pdf]

Cis-acting variation is common across regulatory layers but is often buffered during embryonic development

Swann Floc'hlay*, Emily S Wong*, Bingqing Zhao* ,

Rebecca R. Viales, Morgane Thomas-Chollier, Denis Thieffry,

David A. Garfield†, and Eileen EM Furlong†

Supplementary figures and methods

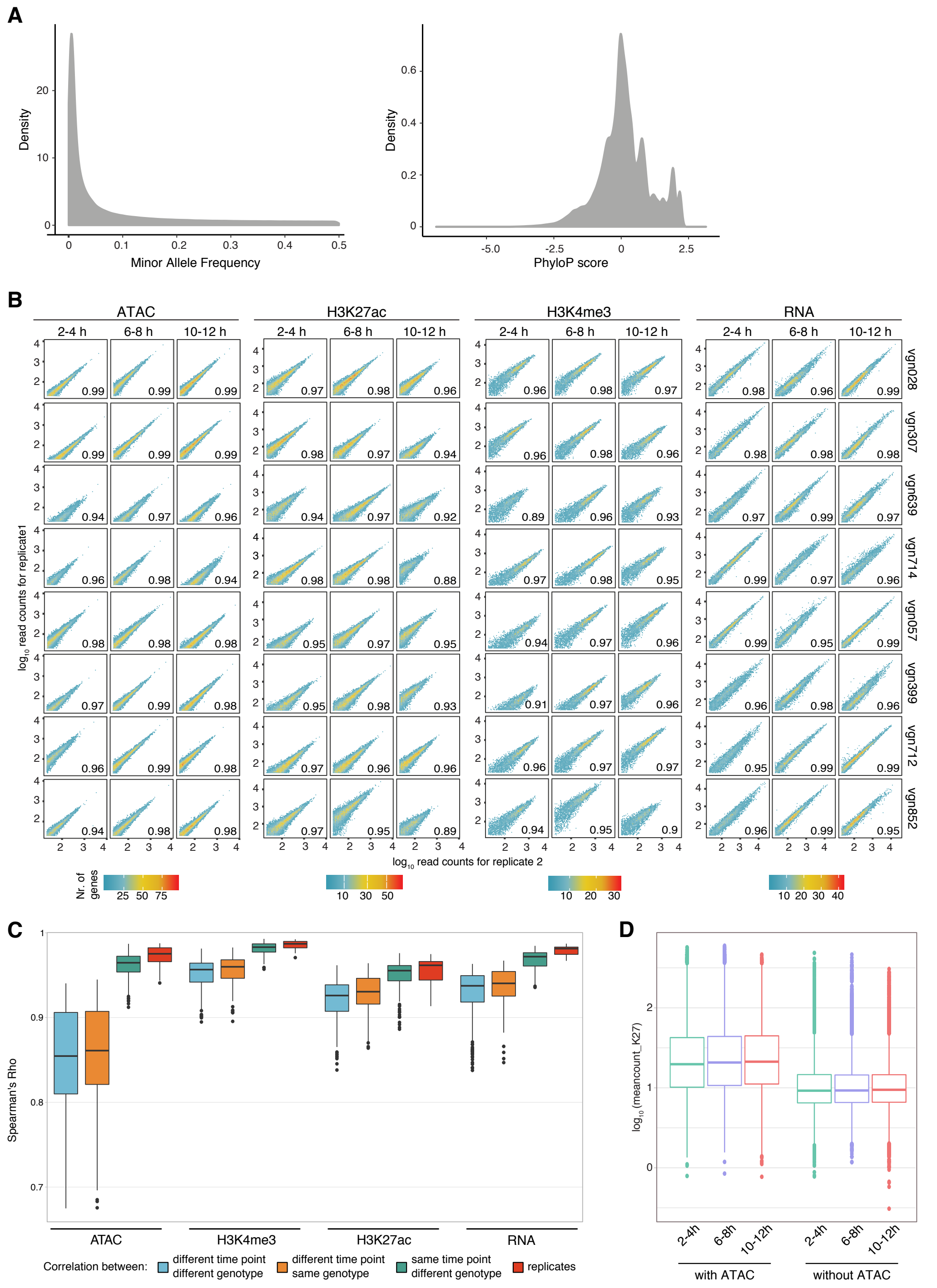

Figure S1: General properties of the data – distribution of SNPs and reproducibility

A. Smoothed histograms show the distribution of minor allele frequencies of SNPs in regulatory elements (left) and regulatory element phyloP scores (right). Regulatory elements are defined here as peaks of ATAC-seq, H3K27ac, or H3K4me3. MAF are taken from the full 205 lines of release 2 of the *Drosophila melanogaster* Genetic Reference Panel. **B.** Gene expression levels and read counts from accessible chromatin/ChIP-seq show consistently high levels of correlation between replicates. Pearson's correlation coefficients indicated. **C.** Box plots comparing the correlations of read count signal between samples from the same or different time points and/or genotypes. **D.** Coverage plots comparing distal peaks of H3K27ac that do (left) and do not (right) overlap annotated ATAC-seq peaks, showing an overall lower read count for the second category.

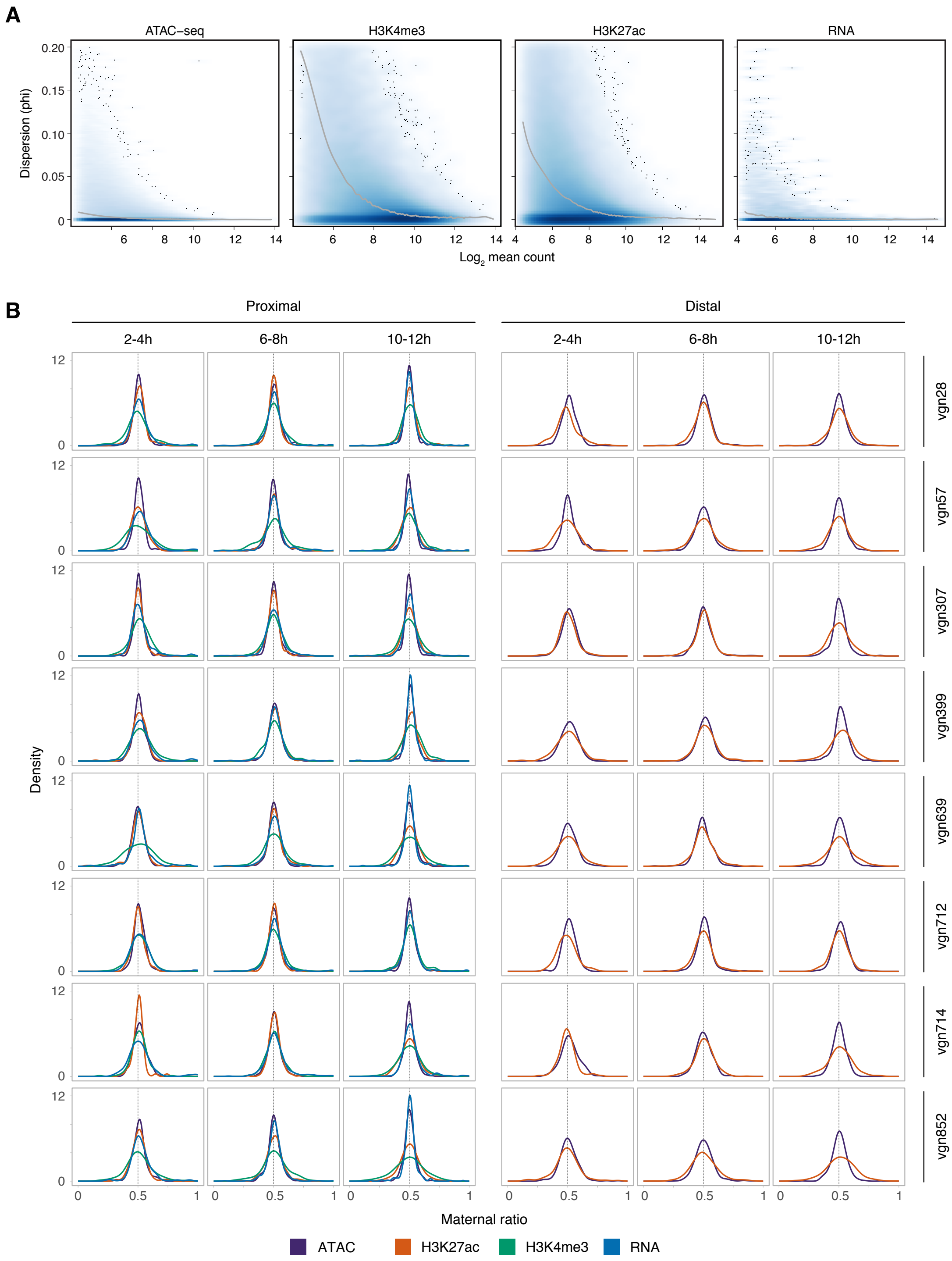

Figure S2: Dispersion and distribution of allelic ratios across data types

A. Dispersion is largely constant as a function of read count for RNA-seq and ATAC-seq. Density plots show the beta-binomial dispersion parameter estimated across our pooled replicates for each feature per time point and per genotype (y-axis) plotted against the log_2 averaged (arithmetic mean) total count across replicates (x-axis). The grey line is a line of best fit. **B.** Density plots showing the distribution maternal ratios for each regulatory layer (colors), at each time-point (columns), for each line (rows), for TSS-proximal and TSS-distal regions separately.

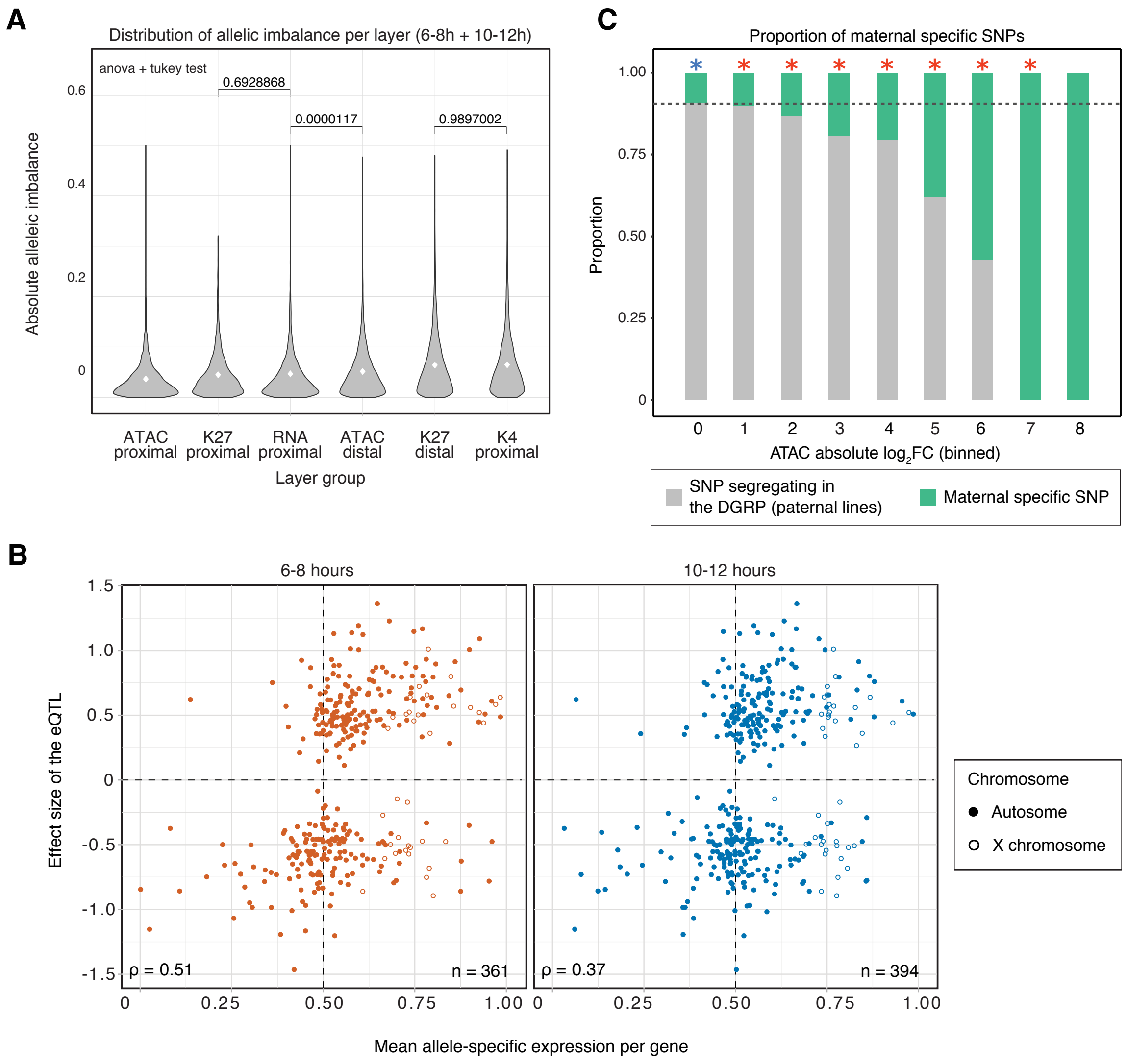

Figure S3: Proportion and distribution of SNPs and allelic ratios

A. Distribution of the absolute departure from allelic balance for each data type. For the same data type, distal features show a significantly higher amplitude of imbalance compared to proximal features (Tukey test, p<0.0001, three highest p-value shown). **B.** Spearman's correlations (ρ) between F1 allelic ratios in autosomes and the effect sizes of associated eQTL identified in Cannavo et al., 2017, show consistent concordance across effect sizes. Allele-specific expression predicts the direction of eQTL in 69% of cases for autosomes (X Chromosomes could not be analyzed here given the inherent bias towards the maternal genotype in F1 pools). RNA-seq data from 2-4 hours were excluded from the analysis due to the presence of maternal transcripts that could bias allelic imbalance towards the mother (right quadrants). **C.** The proportion of SNPs unique to the maternal line compared to any of the DGRP lines (green bars) is greater in highly imbalanced ATAC peaks at 6-8 and 10-12 hours, indicating a correlation between the presence of rare (potentially *de novo*) mutations and large effect sizes. Dotted line shows the average proportion across all the allelic imbalance (AI) values. Green and red asterisks indicate a statistically significant depletion or enrichment, respectively, of maternal specific SNPs for each bin of AI values.

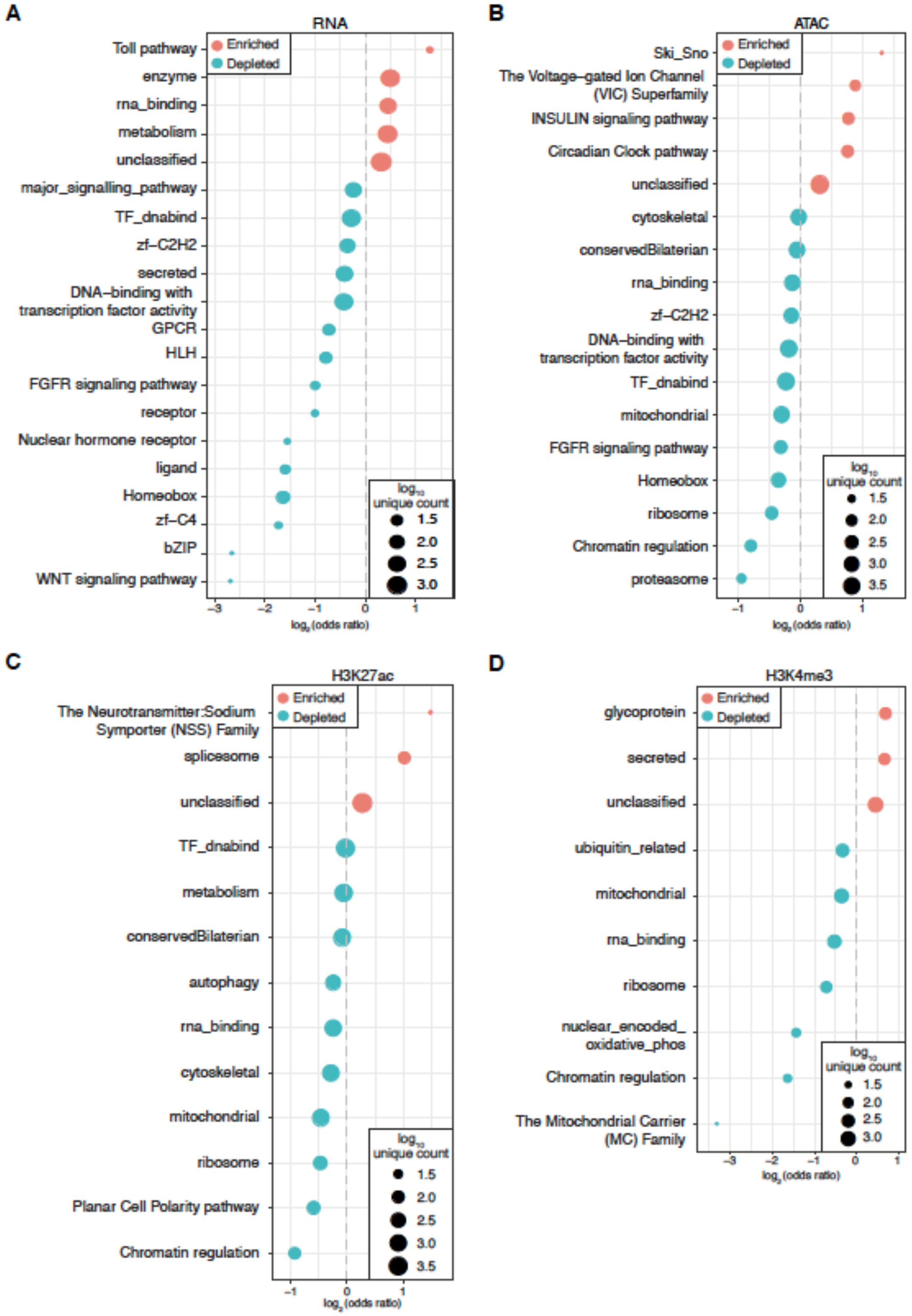

Figure S4: Allelic variation is consistently depleted for transcriptional regulation and enriched for the expression of metabolic genes

Enrichments for all GLAD categories with statistically significant ($p < 0.01$, Fisher's-Exact Test) enrichment or depletion of significant allelic imbalance for **(A)** RNA, **(B)** ATAC or **(C-D)** histone modifications. Bubble size represents the number of genes/features per category. For regulatory elements, category assignments were made on the basis of closest gene annotations.

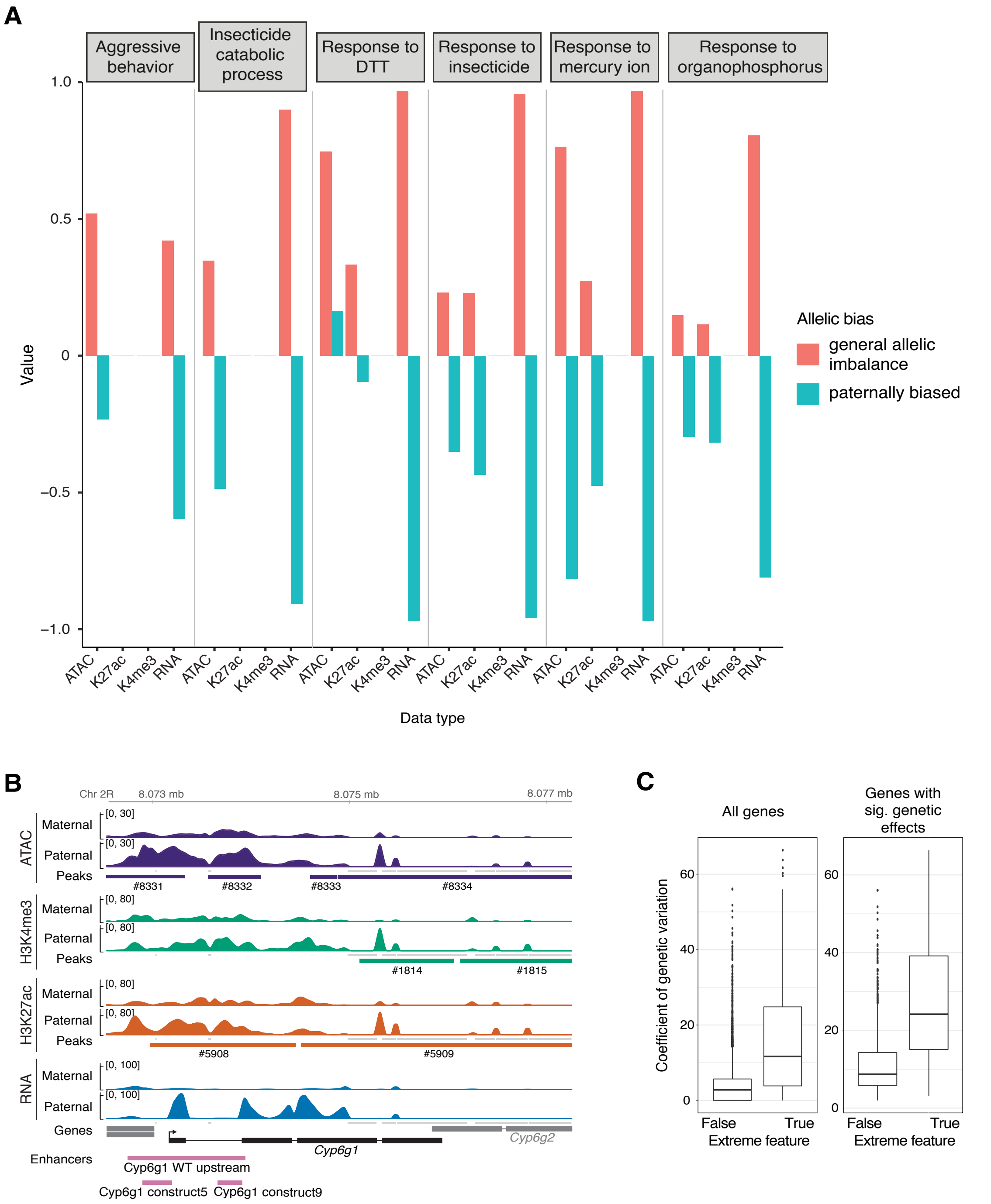

Figure S5: Regulatory changes on paternal haplotypes are enriched for genes related to pesticide resistance and environmental response.

A. Gene categories involved in environmental response and immunity show consistent biases towards the paternal allele. Coefficients from point-biserial correlation analyses are plotted, with red bars indicating correlation with the absolute value of allelic imbalance and blue bars (negative values) indicating correlation with allelic imbalance with the paternal allele being more highly expressed. **B.** *Cyp6g1*, a DTT resistant gene, shows an upregulated paternal allele in every regulatory layer and in gene expression, with the maternal allele showing no evidence of expression (matching reports in FlyBase). Grey bars indicate locations of non-uniform mappability across lines. **C**. Coefficient of genetic variation for highly imbalanced genes vs. all others (left) or only those with significant line effects (right).

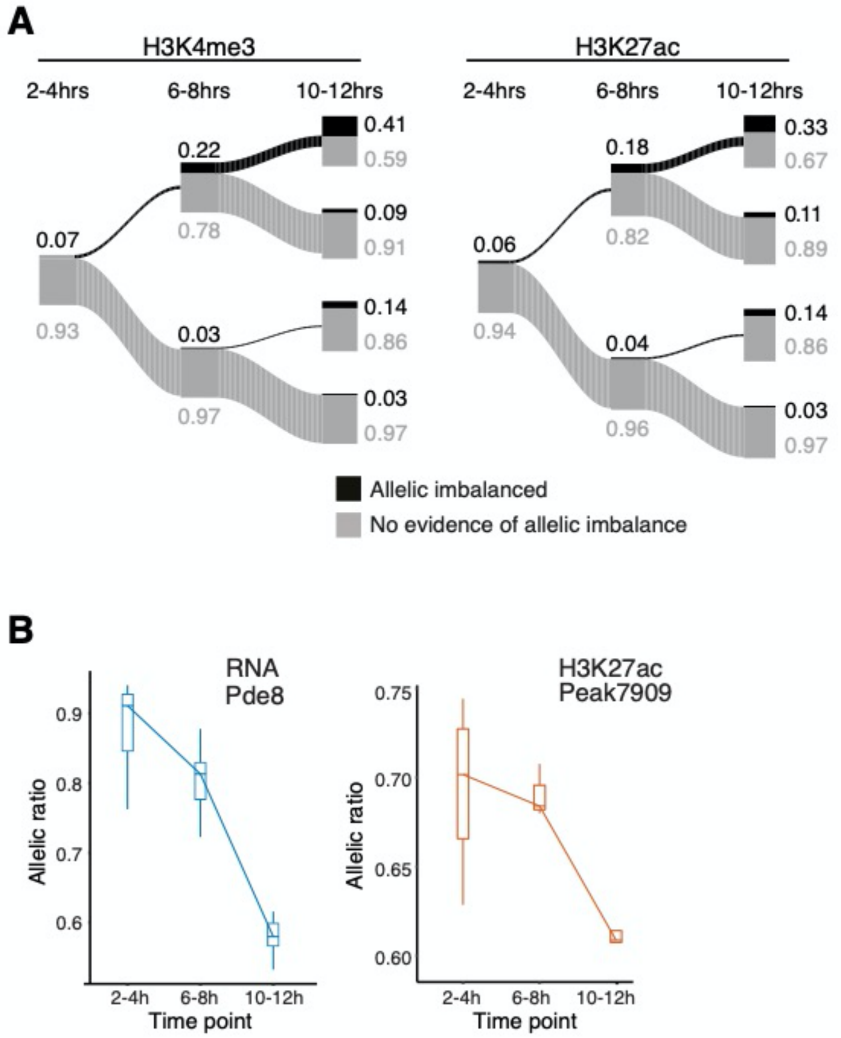

Figure S6: Time and genotype effect over development

A. Flow diagram showing dynamics of allelic imbalance (AI) in chromatin marks across developmental time. Proportions of AI and non-AI features are shown in black and grey, respectively, and represented by the line thickness. Exact proportions for each category are provided as numbers. **B.** Histograms showing allelic imbalance for the *pde8* gene. Allelic imbalance changes across time for gene expression level and associated H3K27ac enrichment.

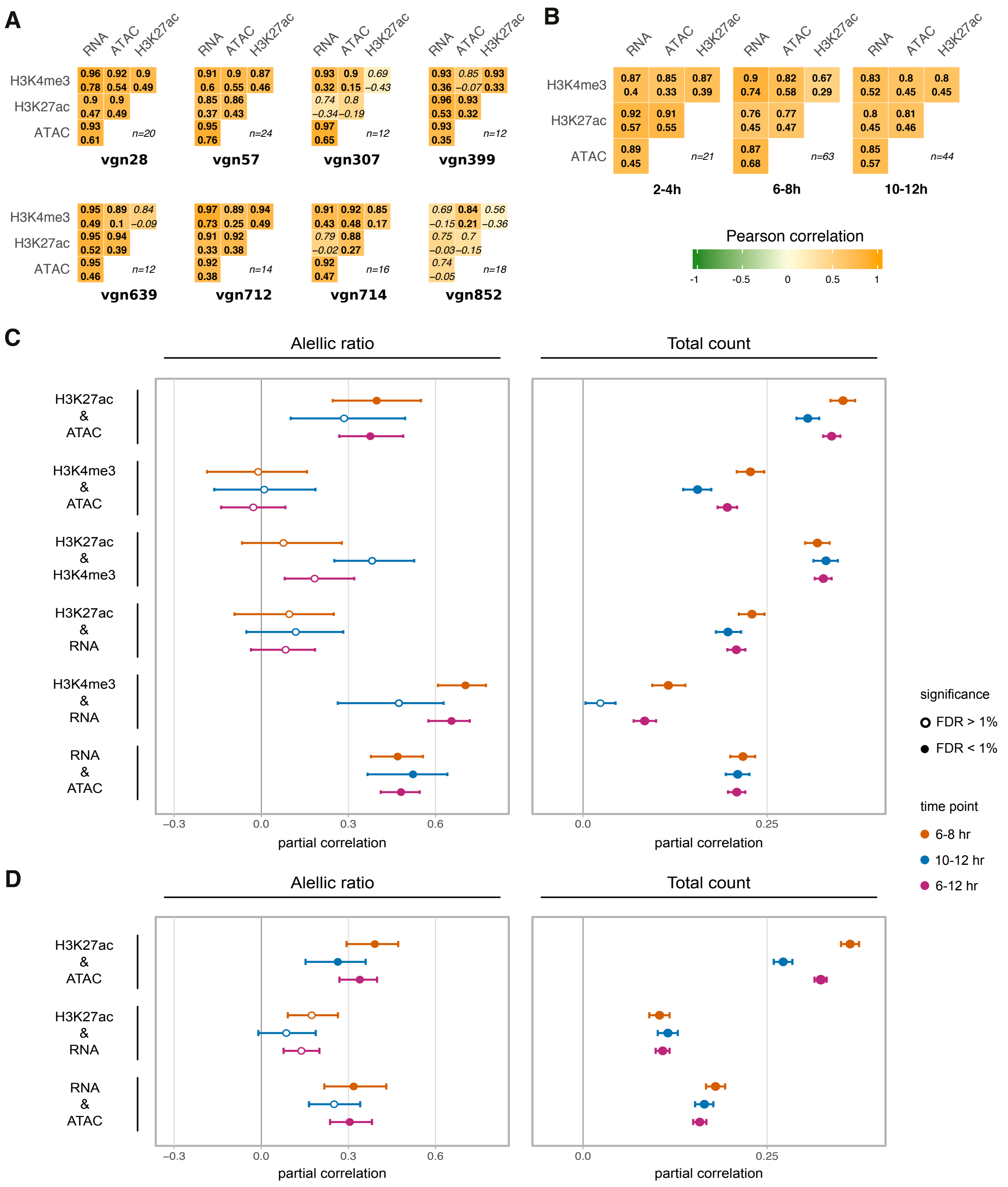

Figure S7: Partial and Pearson's correlations on thresholded allelic ratios

A-B. Heatmaps showing the correlation of allelic ratio between each regulatory layer, grouped by genotype (**A**) and by time points (**B**). Values represent the 5% confidence interval for each pairwise correlation and significant results are emphasized in bold. **C-D.** Partial correlation results for allelic ratio and total count values for TSS-proximal (**C**) and TSS-distal (**D**) features, grouped by time points. Whiskers represent the 95% confidence interval ascertained via bootstrapping. See Supplemental Methods (M3) for the procedure used for thresholding.

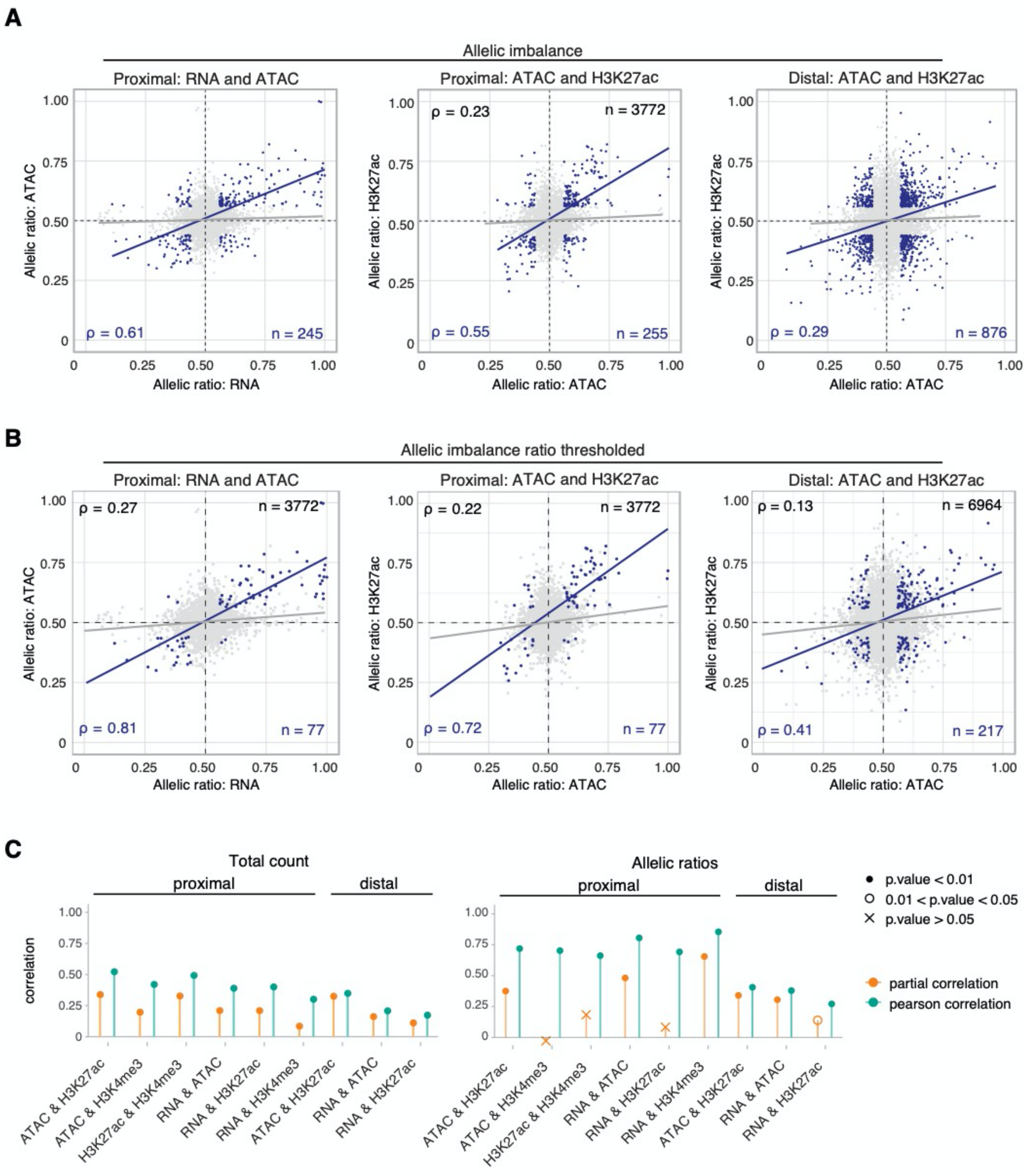

Figure S8: Partial correlation analysis reveals potentially causal relationships among regulatory layers.

A. Overall Pearson's correlations in allelic ratios (black values, grey points and regression lines) between regulatory layers. Correlations are generally modest with little correspondence in allelic ratios between overlapping non-coding features and associated genes. **B.** Correlations in allelic ratios for more imbalanced features (thresholded AI 0.5 +/- 0.06 for all regulatory layers; blue values, points and regression line) are stronger. **C.** Comparison between the partial (orange) and the Pearson's (blue) correlation for total count (left) and allelic ratios (right). A decrease in partial correlation denotes a lack of direct relationship within the overall correlations.

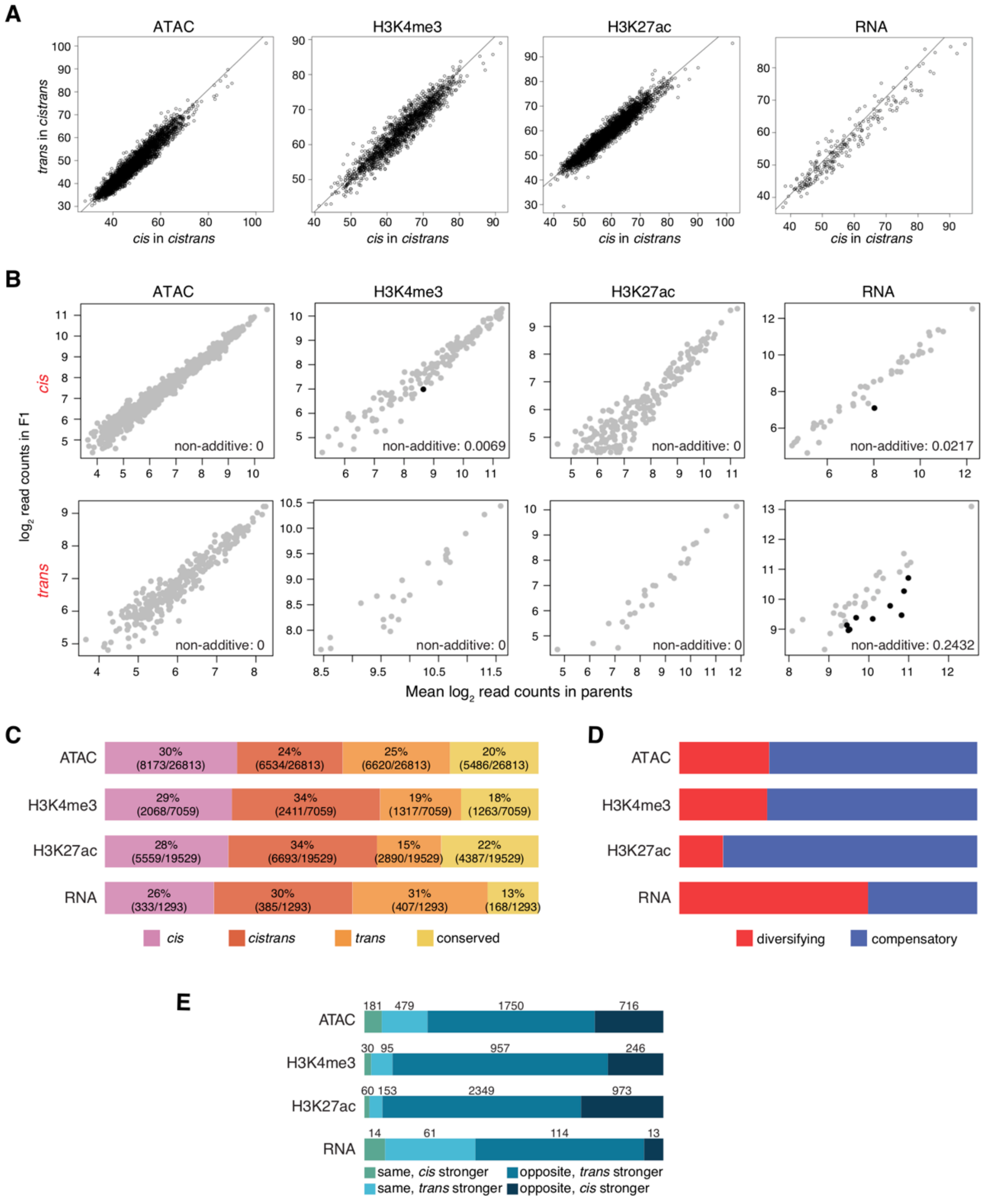

Figure S9: Differences in the frequency of *cis* and *trans* acting genetic variation among regulatory layers influences the heritability of regulatory phenotypes

A. For each regulatory layer, *cis-trans* classified genes/features were assessed to evaluate the relative contributes of *cis* and *trans* to the *cis/trans* signal. Only RNA has evidence for an unequal contribution (more *trans* than *cis*). **B.** Scatterplots showing total read counts in the F1 lines vs. the mean of the two parents for genes/features classified as *cis* (top) or *trans* (bottom). Shown in black are genes/features with an F1 total read count significantly different (FDR <= 0.1) than the parental mean, indicating non-additive heritability. Only *trans*-influenced RNA has a frequency of non-additive heritability meaningfully distinct from 0. **C.** Maximum likelihood *cis-trans* classification composition for each data type (genes and regulatory layers). Numbers and horizontal bars represent the size and relative proportions of each *cis-trans* class in each of the four regulatory layers. See Supplementary Table S8 for a classification of each gene/feature including BIC. **D.** Using the classification scheme of Landry et al, (Landry et al. 2005), we assessed the frequency of diversifying and compensatory evolution for genes and features showing both *cis* and *trans* influences. For regulatory features, the

concordance of *cis* and *trans* effects suggests predominantly compensatory evolution, while RNA shows a pattern more consistent with diversifying selection. **E.** An alternative classification of selective forces for *cis-trans* features following Goncalves et al. (2012), which is designed more explicitly for within species contrasts. As in (d), likely positive selection (*cis* and *trans* working in the same direction) is significantly more common for RNA than for non-coding features.

Supplemental Methods

Fly husbandry, crosses and embryo collection 12
Whole-embryo RNA-seq 13
Whole-embryo ATAC-seq 13
Whole-embryo iChIP 15
Whole embryo gDNA-seq 15
Sequenced reads processing 16
  Personal genome construction 16
  Read mapping 16
  Mappability filter 17
  Quality Control 17
  Peak-calling 19
Total signal quantification 19
  Read assignment 19
  Differential expression across time points 19
  Library size normalization and other processing of total counts 19
Allele-specific signal quantification 20
  Read assignment and gDNA correction 20
  Maternal transcript identification 20
Statistical test for allelic imbalance 21
  Allele-specific changes across lines and developmental time 22
  Gene category enrichment of allelic imbalance 23
Allele-specific changes across regulatory layers 24
  Overlap definition across layers 24
  Partial correlation 26
  Copula directional dependence analysis. 26
  Locus-specific test of gene expression with numbers of open chromatin regions 27
Cis-trans analysis. 27
  Statistical method for steady-state assignment of cis/trans regulatory mechanism 27
  Control for difference in staging between samples 28
Calculating broad sense heritability and coefficient of genetic variation. 29
Measuring additive vs. non-additive heritability 30
The link between *trans* variation and gene category 30
The impact of *cis* and *trans* variation on patterns of selection 31

References 32

Supplemental Methods

Fly husbandry, crosses and embryo collection

The virginizer line was maintained at 20℃ in bottles and flipped once per week. 100-200 adult flies of 5-10 days age were transferred into fresh bottles. The flies were left to lay embryos for about 24 hours at 20℃ before the adults were removed. The bottles were placed inside a special "heat shocking tray" (a fly tray with holes on the bottom and sides) and the tray was placed inside a 38℃ water bath for 1 hour. After removal from the water bath, the bottles were kept at 18℃, 20℃ or 25℃ to synchronize bottles from multiple days. Once the adult flies eclosed, they were flipped into fresh bottles, to be crossed with the males from the paternal line later.

The virgins females that eclosed from the heat-shocked bottles were collected and mated to males from the paternal lines and placed in collection containers at 25℃ two days prior to embryo collections (Sisson 2007). On the day of collection, three one hour prelays were performed to stimulate laying, and thereby clear females of any held embryos, followed by 2-hour collections. The plates with embryos were aged to the appropriate stages at 25℃, followed by washed into sieves and dechorionated by incubating in 50% bleach for 2.5 min. The embryos were extensively washed and blotted dry on tissue paper. Approximately 200 embryos were transferred to an eppendorf tube and snap-frozen for subsequent RNA assays. The majority of embryos were fixed in 1.8% formaldehyde (9.5 ml cross-linking solution with 485 µl 37% formaldehyde) with vigorous shaking for 15 min at room temperature. Cross-linking was stopped by incubating with Glycine solution with vigorous shaking for at least 1 min. After washing with PBT, the embryos were blotted dry. A small aliquot of embryos (100-200) was transferred into a tube containing 0.5 mL heptane and 0.5 mL methanol and shaken vigorously for 2 min to devitellinize the embryos. The embryos were left to settle and liquid was removed as much as possible, followed with 2 washes of methanol and store at -20℃ in methanol for evaluation of the developmental stage of each collection. The bulk of cross-linked dry embryos were transferred into 1.5 mL eppendorf tubes, snap-frozen in liquid N2 and stored at -80℃.

The samples stored in methanol at -20℃ were rehydrated at room temperature by incubating with 50% methanol / 50% PBT for 5 min on a nutator. Nuclei were stained by incubating the embryos in PBT with DAPI for 5 min on a nutator, followed by washing with PBT twice. After removing as much liquid as possible, 80% glycerol was added to the

embryos and left to settle in glycerol and then mounted onto a microscope slide, covered with a cover slip and sealed with transparent nail polish. The developmental stages of the embryo collection were evaluated by examining the morphological features under the Axio Imager M2 research microscope (Campos-Ortega 1997).

Whole-embryo RNA-seq

Embryos were thawed in 200 µl Trizol on ice and smashed with pestle and cordless motor. Another 800 µl Trizol was added to each sample and samples were shaken by hand for 2-3 min, incubated at room temperature for 5 min. 200 µl of chloroform was added and samples were vigorously shaken for 30 sec by hand and centrifuged for 15 min at 12,000g at 4℃. The aqueous phase was transferred to a clean tube and 500 µl isopropanol added to precipitate the RNA by incubating at room temperature for 10 min, followed by centrifugation for 10 min at 12,000g at 4℃. The RNA pellet was washed twice with freshly made 80% EtOH and resuspended in 44 µl nuclease-free water. DNA was digested by adding 5 µl DNase 10x buffer, 1 µl DNase and incubated at 37℃ for 30 min. RNA was purified using RNAclean XP beads. The concentration of total RNA was measured with the Qubit RNA assay kit. If necessary, the size distribution of the library was checked using RNA 6000 Pico kit on the Bioanalyzer.

5 µg of total RNA was taken from each sample and mRNA was extracted using Dynabeads® mRNA Direct Purification Kit, following the manufacturer's manual. RNA tagmentation, reverse transcription, cDNA synthesis and sequencing library preparation were done using NEBNext Ultra Directional RNA Library Prep Kit following the manufacturer's manual.

Whole-embryo ATAC-seq

Frozen embryos were transferred into a 7 mL douncer placed on ice, thawed in 4 mL HB buffer supplemented with proteinase inhibitor cocktail cOmpleteTM. Embryos were dounced 20 times with the loose pestle and 10 times with the tight pestle. The homogenate was filtered through two layers of Miracloth that were positioned 90° to each other in a clean 15 mL tube. The sample was centrifuged for 7 min at 3500 rpm at 4℃ and the murky supernatant was discarded. The nuclei pellet was resuspended in 3 mL HB buffer and transferred to a clean 15 mL tube. The nuclei were dissociated by passing them 10 times through a 20G needle and 10 times through a 22G needle using a 5 mL syringe and then filtered through 20 µm Nitex membrane on a funnel into a clean 15 mL tube.

The nuclei extracted were quantified using the LSRFortessa system. 50 μl diluted nuclei were mixed with 50 μl CountBright Absolute Counting Beads, 197 μl PBS and 3 μl 100x DAPI and loaded onto the LSRFortessa. The nuclei concentration was calculated as:

55000*(nuclei detected/beads number detected)*dilution factor/50 = nuclei/μl.

With the quantification of the nuclei solution, we kept 3 aliquots of 200,000 nuclei in 50 μl PBT for ATAC-seq and 6 aliquots of 40,000 nuclei in 10 μl PBT for iChIP for chromatin marks. The remaining nuclei were centrifuged at 3500 rpm for 5 min at 4℃, supernatant discarded, snap-frozen in liquid N2 and stored at -80℃.

For ATAC-seq, one aliquot of 200,000 nuclei in 50 μl PBT was thawed on ice supplemented with 50 μl permeabilization solution and incubated on a nutator for 30 min at 4℃. The permeabilized nuclei were centrifuged at 3200 g for 5 min at 4℃ and supernatant removed. The nuclei pellet was resuspended in 50 μl tagmentation mix, containing 5 μl TDE1, 25 μl 2x tagmentation buffer (both from Nextera kit) and 20 μl nuclease-free water, and incubated at 37℃ for 30 min. 50 μl STOP buffer and 5 μl of 1 mg/mL RNase A were added to stop the tagmentation reaction and digest RNA by incubating on a thermomixer at 55℃ for 10 min with 900 rpm shaking. The reverse cross-linking and protein digestion were done by adding 3 μl of 20 mg/mL Proteinase K and incubating at 65℃ overnight with 900 rpm shaking. The reaction was purified using a MinElute column with Qiagen PCR purification buffers. The DNA concentration was measured using Qubit. 20 ng of DNA was brought to 10 μl with water. 2.5 μl i7 primer, 2.5 μl i5 primer, 2.5 μl Nextera primer cocktail and 7.5 μl Nextera PCR mastermix (all from Nextera kits) were added to complete the PCR reaction. PCR was run using the following program: 72℃, 3 min; 98℃, 30 sec; cycle start: 98℃, 10 sec, 63℃, 30 sec, 72℃, 3 min, cycle close (run 12 cycles); Hold at 10℃. The PCR product was run on a 1.2% agarose gel supplemented with 1:20,000 GelGreen for ~50 min at 80V. On the gel, every two samples were separated by one empty lane to avoid cross contamination. Gel piece from right above the primer dimer band (~100 bp) to 1 kb was cut with SafeXtractor (one-time use only) on a blue light transilluminator. DNA was extracted from the gel using Qiagen gel extraction kit and eluted with 2 MinElute columns for each sample. The concentration of the libraries was measured with Qubit, and the 8 samples were multiplexed in equal molarity and sequenced on Illumina HiSeq 2500 platform to obtain 125 bp paired-end reads.

Whole-embryo iChIP

iChIP experiments were performed as described in Lara-Astiaso et al. 2014 (Lara-Astiaso et al. 2014). In brief, 40.000 *D. melanogaster* nuclei in a total volume of 10 µl PBS + 0.5% SDS were used per sample. We usually processed 24 samples in one iChIP experiment. The nuclei were sonicated in a 0.1 ml sonication tube (Diagenode Cat. No. C30010015) at 4°C for 40 cycles 30''/30'' ON/OFF using the Bioruptor Pico (Diagenode). For the first IP, Chromatin was immobilized with 1.3 µg of anti-H3 antibody (abcam ab1791) and Dynabeads Protein G (Invitrogen, Cat. No. 10003D) on a rotating wheel overnight at 4°C. Following Chromatin End Repair and A-tailing, the samples were indexed by ligation of Y-shaped Index Adaptors containing P5 and P7 sequences.

After Chromatin Release, samples were pooled by 8, resulting in three pools. These pools were split into two second IPs each. The chromatin was incubated at 4°C for 3h on a rotating wheel with 2.5 µg of antibody against H3K27ac (abcam, ab4729) or H3K4me3 (Millipore, 07-473), followed by a 1 h incubation with Dynabeads Protein G. The IPs were washed, the ChIPed DNA was eluted and purified. For Library Amplification, KAPA HiFI Hot Start Ready Mix (KAPA Biosystems, KM2605) and primers containing the Illumina P5-Read1 and P7-Read2 sequences were used. After a final clean-up with AmPure XP beads (Beckman, Cat No. 63880), the quality of the libraries was determined by qPCR. To assess efficiency and specificity of the ChIP, we used a positive and a negative target region and compared the enrichments by qPCR before and after library amplification. The libraries were run on a Bioanalyzer chip, multiplexed and sequenced with Illumina NextSeq500 high 150bp PE.

Whole embryo gDNA-seq

We isolated gDNA from ~100 embryos per F1 cross using an electric pestle and Solution A from Qiagen (0.1mM Tris-HCl pH9.0 / 0.1 M EDTA / 1% SDS). Following this step, the lysate was incubated for 30 min at 65˚C followed by the addition of 28 µl 8M KAc and 30min incubation on ice. The debris was then spun down and the supernatant cleaned up using a standard Phenol/Chloroform extraction. The resulting gDNA was processed in a 75bp SE sequencing library using NEBNext DNA Ultra 2 protocol on a Hamilton automated Liquid Handling system and sequenced on an Illumina NextSeq.

Sequenced reads processing

Personal genome construction

As a starting point for all personal genomes, we began with *Drosophila* reference assembly dm3 as downloaded from the UCSC genome browser (version 5 from FlyBase) along with reference annotations r5.57 from FlyBase. To generate reference genomes for our paternal lines, we downloaded variant calls for the full 205 lines of the DGRP (http://dgrp2.gnets.ncsu.edu/) made against the dm3/v5 *D. melanogaster* reference genome. For each paternal line from DGRP, we inserted SNPs and indels from the VCF file into the reference genome. Changes in coordinates were recorded in a liftOver chain file for subsequent steps. Heterozygous SNPs were replaced with the appropriate ambiguity code with missing data ('.') recorded as an N. Heterozygous indels were inserted as a string of N equal to the length of the longer haplotype. In the case of the Virginizer line, we made use of a VCF file generated for reference genome dm6/v6 (Ghavi-Helm et al. 2019) and converted the coordinates to dm3 using pslMap (http://genome.ucsc.edu/, v5). The same steps for reference generation was used for the DGRP paternal lines. For all parents, a genotype-specific set of annotations was created using liftOver to translate the r5.57 reference GFF3 file into the coordinate frame of the custom parental genome.

Read mapping

Read were trimmed for adaptor sequences and sequencing quality using skewer (v0.2.2) and seqtk (v1.0), respectively, with default parameters. To avoid mappability bias, we used the parental genome mapping strategy (see mappability filter section, below) and mapped reads to both personalized parental genomes (Skelly et al. 2011). Reads from ATAC-seq and ChIP-seq were mapped using BWA (v0.7.12) (Li and Durbin 2010), reads from RNA-seq were mapped using STAR (v2.5.1b) (Dobin et al. 2013) and FlyBase gene annotation version 5.57. Aligned reads were clipped when overlapping their read pairs using clipOverlap (v1.0.14). Using the appropriate chain files and pslMap (http://genome.ucsc.edu/), alignment coordinates were converted into the reference *Drosophila melanogaster* r5.57 genome coordinate space, also used in the DGRP project for variant calling.

Resulting alignments from both parental genome mappings were merged into a single alignment file, where reads aligned in both cases were reported only once (selecting the alignment with the highest mapping score).

Mappability filter

To ensure equal mappability across the two parental genomes for a given F1 line, we made use of two approaches. First, genomic DNA sequencing data from all the parental lines were mapped using STAR on their personalized genome and converted into the r5.57 reference using pslMap (http://genome.ucsc.edu/). Coverage data was produced using pslToBed (http://genome.ucsc.edu/) from the UCSC genome browser utilities. For each of our F1 crosses, genomic regions showing a null coverage in either the mother or the father line were discarded from the analysis by trimming the portion of the aligned reads overlapping such regions. Second, for each of the parental genotypes, we simulated transcriptomic and genomic reads spanning the entire genome with equal coverage (one read starting at each base pair, read length=100bp). The resulting reads were mapped on the corresponding parental genome and converted to the reference genome coordinates in the same manner as the RNA-seq, ATAC-seq and ChIP-seq experiments. For each of the F1 lines, regions showing a different coverage between the paternal and maternal synthetic read mapping were not considered when calling allele-specific measures. This step captured mappability issues caused by ambiguous bases and Ns introduced during the construction of the parental reference genomes. In order to compare total coverage measures across samples, a universal mappability filter encompassing all the line-specific filtered regions was applied to trim the reads before further analysis.

Quality Control

We evaluated the quality of our sequencing data in three ways. First, we looked at pairwise correlations between each sample. We observed a Spearman's correlation of at least 0.95 in all biological replicates (Supplemental Fig. S1b), while the median Spearman's correlations between samples from different genotypes or different time points were lower, as expected, ranging between 0.84 and 0.98 for all data types (Supplemental Fig. S1c). Second, we performed a principal component analysis at the level of total counts to look for evidence of issues for specific samples (e.g. failure to cluster with a replicate or clustering with the wrong time point). Third, in the case of RNA, we looked for correlations between our samples and the modENCODE time series of development (Supplemental Fig. M1, below) (Graveley et al. 2011). Through these last two steps, we realized significant issues with the 6-8hr time point for the parental line 399 – while the replicate correlations are high, these samples appear closer to 10-12hr than they do 6-8hr. We thus removed these two samples

from all analyses, reducing our *cis*/*trans* analyses for RNA to only the 10-12hr time point. No similar staging issues are apparent in the open chromatin or histone modification data.

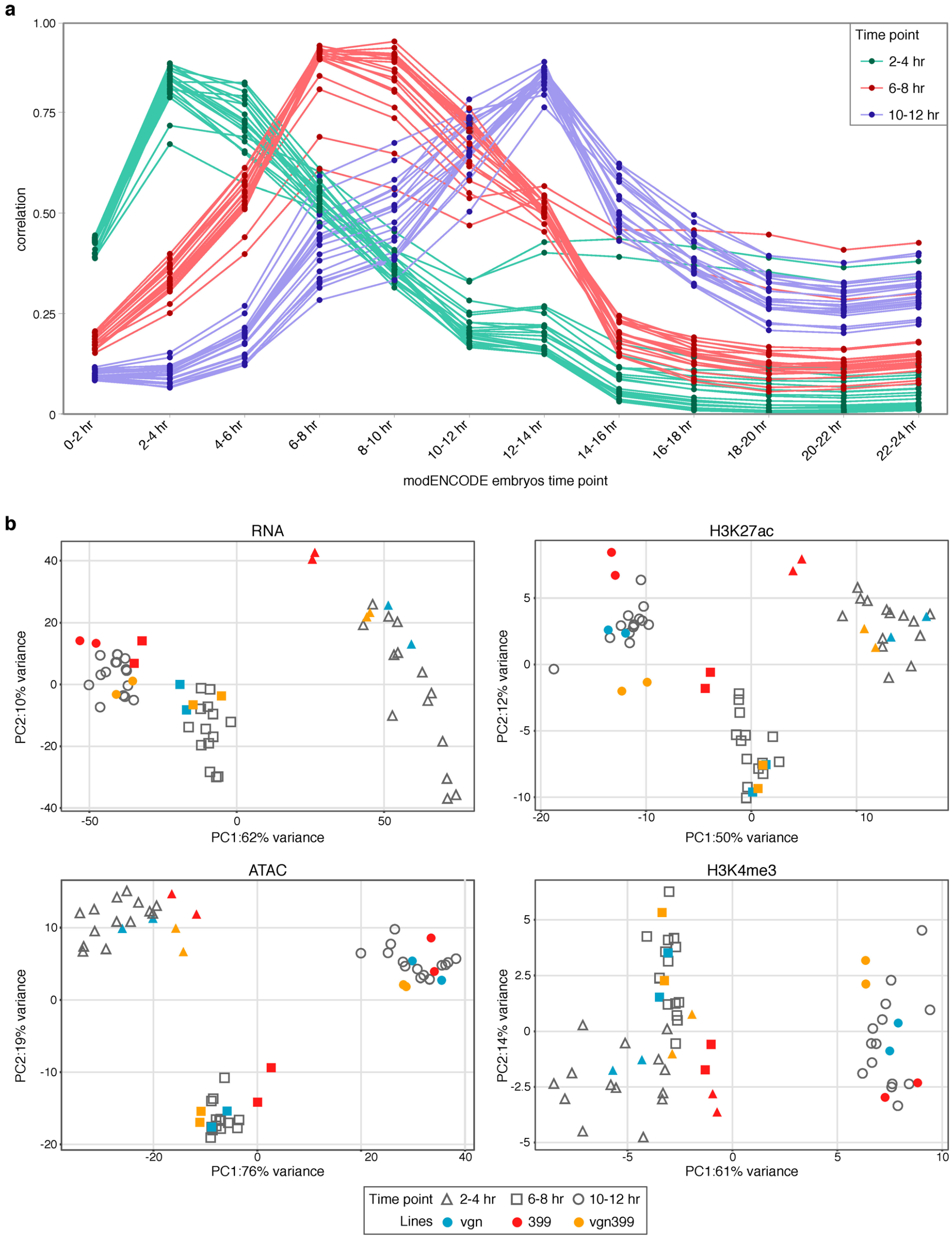

Figure M1: Total count data cluster with similar time points

a. Correlation of gene expression total count data with modENCODE time series. As expected, the highest correlation corresponds to the comparison with equivalent time points. **b.** Principal component analysis of total count data for each data type. Parent-offspring trio is shown in color. For gene expression data (upper, left), the two replicates of parental line 399 at 6-8hr cluster together with the 10-12hr samples. We removed these samples from further analysis.

Peak-calling

For ChIP-seq experiments targeting H3K4me3 and H3K27ac marks, peak calling was performed for each sample on each parental genome using Macs2 (v2.1.1) (Zhang et al. 2008) with the broad option and default parameters. After converting all peak calls to the dm3 coordinates, we merged peaks using the bedtools merge function to produce a single peak set used across all lines and all developmental time points (Quinlan and Hall 2010). For ATAC-seq experiments, regions of chromatin accessibility were defined as the merge of peak summits called by Hotspot (v4.0.0) (John et al. 2011), with a score higher than 5 in at least one of the F1 samples after extending the summits by 200bp in both directions.

Total signal quantification

Read assignment

For each individual sample, total coverage signal was evaluated at the feature level (genes or peaks) using scripts accessing the pysam package (Li et al. 2009). Each read mapping to at least one of the two parental genomes and not filtered by the mappability filter was assigned to its overlapping feature. Reads not overlapping a SNP were also included in this process, as this measure is not allele-specific.

Differential expression across time points

To quantify changes in total read counts between time points, we imported the total count data into DESeq2 and fit a model consisting of line + time (Love et al. 2014). For any gene with a significant (FDR < 0.1) time effect, we subsequently looked for evidence of changes between neighboring time points using the contrasts option in DESeq2. One concern is that some genes may have expression levels that are simply too low to provide statistical power. To avoid this issue, we presented results only from genes whose mean expression across all three time points was equal or greater to the read count threshold identified via DESeq2 implementation of independent filtering (Bourgon et al. 2010).

Library size normalization and other processing of total counts

Upon visual inspection of the total count distribution across data types, we set the minimum of 20 reads per feature as the threshold for detecting expressed genes and non-coding peaks. Total counts were library scaled and TMM normalized using EdgeR (Robinson et al. 2010). Values are expressed in log10 (Counts Per Million).

Allele-specific signal quantification

Read assignment and gDNA correction

For each dataset, allele-specific counts were performed at the feature level, i.e. per genes for RNA-seq and per peak for ATAC-seq and ChIP-seq. Based on their genotype at the SNP location, reads overlapping a feature were assigned to the maternal or paternal haplotypes. Reads not overlapping a segregating SNP or reads with disagreeing assignment between SNPs were ignored in the measurement. Cases of genotyping errors can potentially lead to incorrect allelic imbalance measure if a SNP is wrongly called as segregating in a given cross. To correct for these events, we performed a genomic DNA-seq experiment for each of the F1 lines and processed it in the same manner as the ATAC-seq data. Using a two-sided binomial test with false discovery rate correction, we tested for each SNP whether the number of reads assigned to the maternal and the paternal haplotypes were significantly different from an expected 50:50 ratio in the autosomes. In chrX, the expected ratio was empirically measured from the 1000 SNPs with the highest coverage in chrX. Only SNPs with a minimum coverage of 15 reads for autosomes and 10 reads for chrX were tested. SNPs considered as significantly imbalanced ($p < 0.05$) for the genomic DNA data were formatted as missing data (N) for alignment and were ignored when performing the allele-specific measures. In order to evaluate the sex ratio of our pool of embryos, allele-specific counts of gDNA reads were also performed at the feature level.

Maternal transcript identification

Due to the presence of maternally deposited transcripts, especially in the early 2-4hr time-point, a portion of the genes has an allelic imbalance biased toward the mother in the RNA-seq data. In order to detect them, we used RNA-seq data from unfertilized eggs from the same developmental time windows as the F1 samples. To identify genes with maternal deposition, we plotted the log10 read count of each gene as measured in freshly laid eggs (the first time window), and used the bimodality of this distribution to set a threshold for "expressed". The majority of these genes were excluded from subsequent analyses. However, as previously noted (Thomsen et al. 2010), we observed a population of these transcripts that decayed over time, becoming not detected by 6-8 hours. Formally, we quantified this population as those transcripts showing significant evidence of decay (using DESeq2) between 2-4h and 10-12h (Supplemental Fig. M2, below). As this population of transcripts shows a 50:50 autosomal ratio in the 6-8h and 10-12h datasets, we included them in our analyses.

In addition to maternal transcript removal, as we used a poly(A)$^{+}$ selection step in the construction of our RNA-seq libraries, any genes that largely or entirely lack polyadenylation signal (e.g. ncRNA, snRNA, rRNA) were removed from our analyses categories.

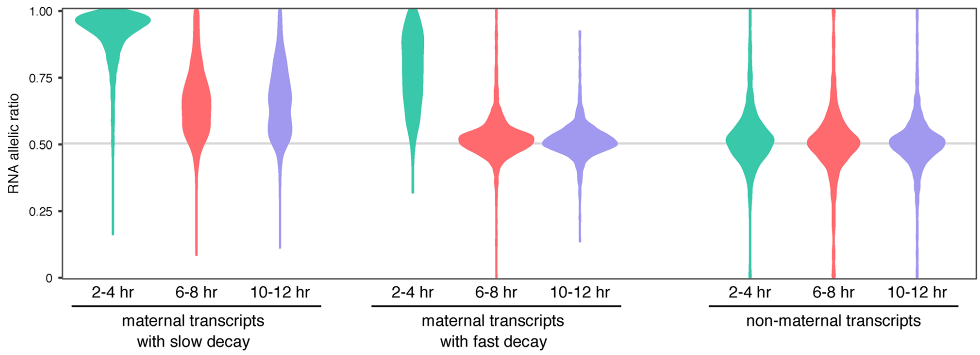

Figure M2: Maternally deposited transcripts show different rates of decay

Distribution of allelic ratios for gene expression data for line vgn28 at each time point. The genes are separated into three categories, based on their expression in freshly laid eggs (i.e. purely maternally deposited transcripts). **Left:** genes detected in unfertilized eggs (maternal transcripts) showing no evidence of decay at 10-12h. **Middle:** genes detected in eggs and showing significant decay between 2-4hr and 10-12h. **Right:** genes not detected in eggs, only zygotically expressed. Zygotic genes and maternally deposited genes with evidence of fast decay are included in the analysis.

Statistical test for allelic imbalance

Individual tests were performed for each line and for each time point. Total F1 counts ($n_g^{s,i,t}$ can be modeled on an allele-specific basis ($z_g^{s,i,t}$) using a beta-binomial distribution. Specifically, $z_g^{s,i,t}$ denotes the number of reads from the maternal allele mapped to feature f for pool of individuals i, of paternal strain s, at time t. $n_g^{s,i,t}$ denotes the total number of reads mapping to genes for pool of individuals i of strain s, at time t.

$$z_f^{s,i,t} \sim Bi(n_f^{s,i,t}, p_f^{s,i,t})$$

$$p_g^{s,i,t} \sim Be(\alpha, \beta)$$

where α, β are the shape parameters of the beta distribution. We tested the following scenarios by maximum likelihood estimation:

$$No\ imbalance: \alpha = \beta$$

$$Allelic\ imbalance: \alpha \neq \beta$$

Due to limited replicates per condition, we used information across features to reduce the uncertainty of estimates and improve power by assuming that all features have the same mean-variance relationship (Robinson et al. 2010; Love et al. 2014). Empirical data was used to estimate the over-dispersion parameter (ρ) for each data type based on the beta-binomial distribution. Maximum likelihood estimation was used to obtain α and β for each feature of time *t* and strain *s*. ρ is calculated as follows:

$$\rho = \frac{1}{\alpha + \beta + 1}$$

The mean over-dispersion value for all features was used as the shrinkage term and likelihood ratio tests (df=1) were used to obtain a p-value, which was adjusted using FDR (Benjamini 1995). Autosomes were tested separately to sex chromosomes; features on the X Chromosome were tested using a background allelic ratio of no imbalance centered on the averaged ratio of maternal versus paternal alleles across the data set being compared (i.e. RNA, ATAC, H3K4me3, H3K27ac). Autosomal features were tested using a null distribution of 0.5.

Allele-specific changes across lines and developmental time

We use a linear mixed-effects model where a random effect is incorporated to estimate variability between strains. Specifically, y_f^{dsr} represents the proportion of reads $\frac{maternal}{maternal+paternal}$, mapped to a feature *f* in data type *d* of strain *s,* replicate *r* and time *t*. Random effect components were incorporated to estimate variability between pools of individuals, time points and lines.

$$y_f^{d,s,r,t} = \mu_f + \delta_f^t + \omega_f^s + (\delta\omega)_f^t \qquad \omega_f^s \sim \mathrm{N}(0, \sigma_f^2)$$

μ_f is the intercept term. δ_f^t is a random effect term denoting time. ω_f^s is a random effect based on strain and $(\delta\omega)_f^t$ is a interaction term for time by strain.

$$M_0: \mu_f \neq 0, \delta_f^t \neq 0, \omega_f^s \neq 0, (\delta\omega)_f^t = 0$$

$$M_1: \mu_f \neq 0, \delta_f^t = 0, \omega_f^s \neq 0, (\delta\omega)_f^t = 0$$

$$M_2: \mu_f \neq 0, \delta_f^t \neq 0, \omega_f^s = 0, (\delta\omega)_f^t = 0$$

$$M_3: \mu_f \neq 0, \delta_f^t \neq 0, \omega_f^s \neq 0, (\delta\omega)_f^t \neq 0$$

To infer the significance of time or strain dependent allele bias, we restrict the values that the parameters can take where $M_0$is the full model that controls for effects due to time, genotype

as well as the time by genotype effect. M_1 is a model where we assume no allelic balance between time points after controlling for strain effects. Conversely, M_2 accounts for allelic imbalance changes by time points while controlling for strain effects. M_3 specifies a non-zero interaction term between time and line. Each model is fitted to the data in turn by maximizing the likelihood using the R library 'lme4' (Bates 2015). Akaike information criterion (AIC) is used to assess the best model.

Prior to analysis, count data filtered for reads with more than 20 allele-combined counts. Additionally, maternally deposited genes were removed for gene expression counts, as described above. For this analysis, the sex chromosome was removed. Each feature is tested using read counts at SNPs common to all lines.

Gene category enrichment of allelic imbalance

To better understand the biological functions affected by *cis*-regulatory variation, we looked for the enrichment/depletion of allelic imbalance in functional categories using a Fisher's exact test. We focused on the gene-centric GLAD categories (Hu et al. 2015), which are broadly representative of the trends observed using other ontologies (Supplemental Table S5). In these analyses, chromatin features (ATAC and histone mark peaks) were assigned to the closest gene, though similar results were obtained if we limit our analysis to only promoter-proximal elements (<500bp from the assigned TSS; Supplemental Table S5).

With this analysis, we observe an enrichment of allelic imbalance in a set of genes and associated non-coding features that could not be assigned to any known GLAD category. Such set collectively represents fast-evolving and *Drosophila*-specific genes, referred as the 'unclassified' category (Mi et al. 2003; Turner et al. 2008) (Supplemental Fig. S4). Enrichment analyses themselves were carried out using Fisher's exact test (for binarized data) or a point-biserial correlation, i.e. a Pearson's correlation coefficient for circumstances in which one variable is continuous and the other categorical (to ensure robust results, point-biserial correlations were also compared to non-parametric rank-biserial correlation analyses). In all enrichment analyses, features/genes from both the X Chromosome and autosomes were included. Similar analyses were performed on evolutionary rate data (Supplemental Table S5) and the heritability measures H^2 and the coefficient of genetic variation.

Allele-specific changes across regulatory layers

Overlap definition across layers

Each set of non-coding features were split into TSS-distal and TSS-proximal subsets. Features are considered as part of the TSS-proximal set if their nearest TSS is not further than 500bp away or if they overlap a region called as a H3K4me3 peak. For each subset, we defined regions of overlap between the regulatory layers as the overlap portion of two or more non-coding features. In the case of the overlap of three features, at least one base pair must be shared with all the layers to create an overlap.

For the proximal subset, genes features are assigned to a given overlap region if the distance between the overlap boundaries and the TSS is smaller or equal to 500bp. For the distal subset, overlaps are associated with the gene having the closest TSS. To avoid mis-assignment of TSS to proximal *cis*-regulatory overlaps, we excluded TSS positioned in the 600bp upstream region of other TSS.

During our partial correlation (and related) analyses, as we noticed a clear drop in the correlation between the regulatory layers when the distance between the nearest TSS and the chromatin features exceed 1500bp (Supplemental Fig. M3a, below), we thus restricted the TSS-distal set to a maximum distance to TSS of 1500bp.

The characterization of overlapping regions (i.e. regions of overlap between multiple non-coding features) resulted in the definition of TSS-distal ATAC-only and H3K27ac-only peaks, which did not overlap each other (Fig. 1c). We assessed the enrichment for regulatory signatures and transcription factor binding motifs of these peak sets using i-cisTarget (Imrichova et al. 2015) and RSAT (Nguyen et al. 2018) (Supplemental Table S3). Strong enrichment for Polycomb and Su(Hw) ChIP-seq signal was observed in ATAC-only peaks (NES > 5), which was further supported by the enrichment for Su(Hw) binding motif in the *de-novo* motif discovery analysis (e-value = 3×10^{-11}). In H3K27ac-only peaks, strong enrichment for Polycomb, Elav and H3K27me3 ChIP-seq signal was observed (NES > 5).

In both ATAC-only and H3K27ac-only peaks sets, an enrichment for RNA polymerase II ChIP-seq signal was also observed (mean NES of 10.26 and 6.02 for ATAC-only and H3K27ac-only peaks, respectively), although it did not reach the enrichment score observed in the overlapping peaks (mean NES = 10.81 and 10.2 for ATAC & H3K27ac overlapping peaks, respectively).

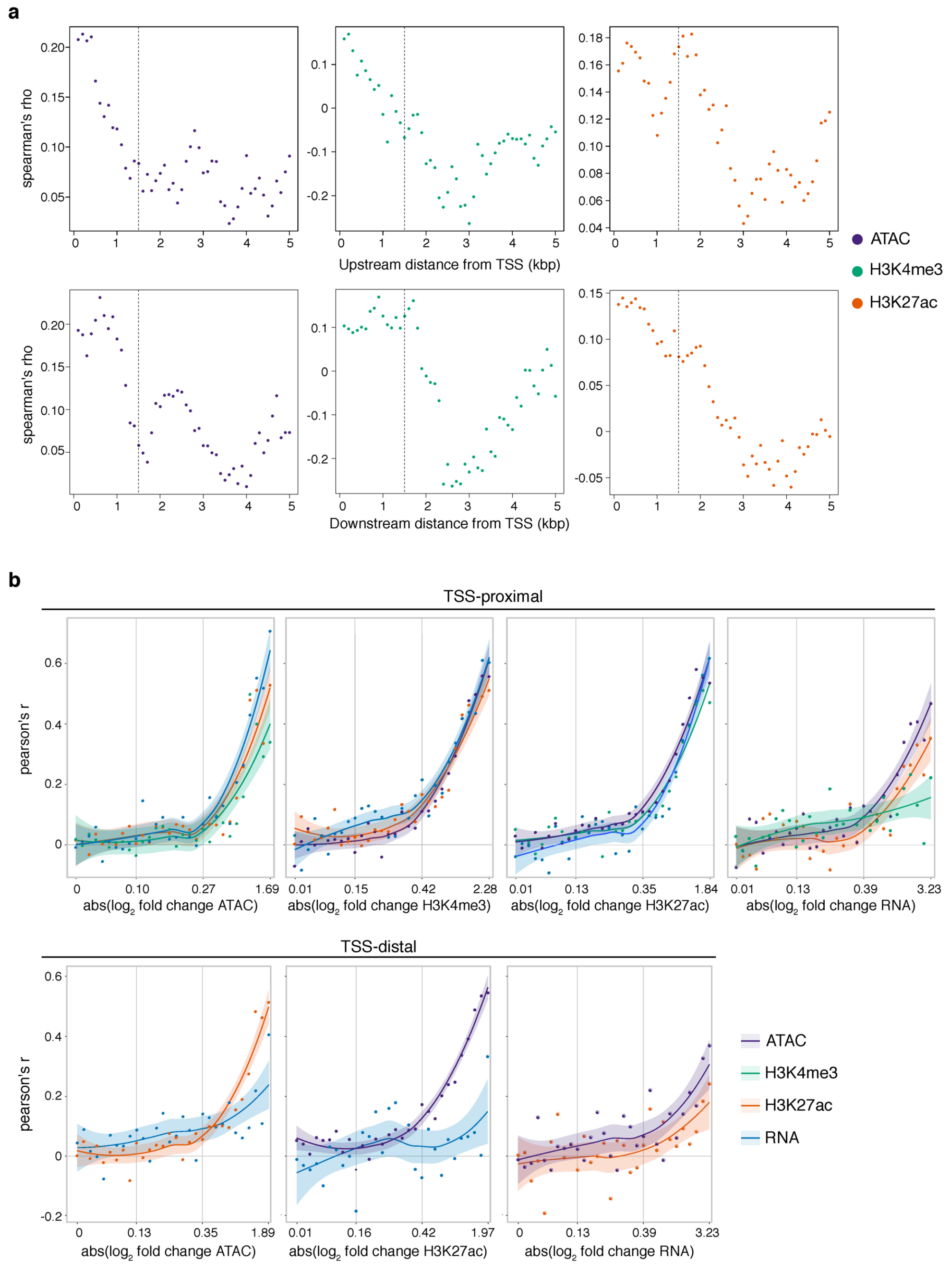

Figure M3: AI and TSS distance thresholding increases correlation between regulatory layers

a. Spearman's correlation of allelic ratios between genes expression and the other regulatory layers, as a function of their distance from TSS. Correlations are shown for non-coding features located from 0 to 5kb upstream (upper row) or 0 to 5kb downstream (lower row) of the TSS. As the correlation values show a clear drop for distances further than 1.5kb, we removed features greater than 1.5kb from TSS (upstream and downstream) from the TSS-distal overlap set. **b.** Pearson's correlation of allelic ratios between regulatory layers for TSS-proximal (upper row) and TSS-distal (lower row) features at 10-12hr. Correlations are binned into 30 quantiles based on the absolute $\log_2$ fold change of allelic ratio. Values on x-axis show the mean $\log_2$ fold change in the 1st, 10th, 20th and 30th quantiles from left to right. Shaded regions indicate the 90% confidence intervals. In most cases, we see an inflection point around the 18th quantile, which was used to set the AI threshold of 0.5 +/- 0.06 in further analysis.

Partial correlation

Partial correlation analysis was performed using the R package 'GeneNet' (Opgen-Rhein and Strimmer 2007) for both allelic ratios and total count data and for TSS-proximal and TSS-distal sets (excluding the X Chromosome). We used features with no missing data in any of the regulatory layers (Supplemental Fig. S8). For allelic ratio data, we observed a distinct increase in Pearson's correlations between layers as the AI fold change increased, suggesting a threshold below which allelic imbalance was effectively "noise". To establish a filtering threshold for "noisy" allelic imbalance, we separated each dataset into 30 bins of total allelic imbalance and plotted the average correlation in allelic fold change between datasets for each bin (Supplemental Fig. M3b, above). In each case, there is a general inflection point in the correlation at an allelic ratio of ~0.5 +/- 0.06. We filtered loci that fell below this threshold to improve the covariance signal of AI datasets for partial correlation analysis (Supplemental Fig. S8).

For both TSS-proximal and TSS-distal analysis, the Pearson's and the partial correlation results are largely consistent between time points and genotypes (Supplemental Fig. S7). Samples from different time points and genotypes were thus pooled for this analysis. Because the different ratios for autosomes and X Chromosomes would lead to an artificially inflated correlation, X Chromosome genes/features were removed for this analysis. We additionally performed bootstrapping over 2000 iterations and 80% sampling to compute confidence intervals (Supplemental Fig. S7).

Copula directional dependence analysis

Directional dependence modeling was done in a regression framework using copulas to describe the bivariate distribution between our pairwise datasets. A copula is a multivariate distribution where the marginal distributions are uniform (Sklar 1973; Kim 2017). Any multivariate function, F(x,y), can be represented in a copula as a function of its marginals, C($F_X(x), F_Y(y)$). Hence, given two random variables X and Y, the copula C(u,v) reflects this dependency, and $U = F_X(x), V = F_Y(y)$ are the marginal variables with uniform distributions. Given an asymmetric copula, it is possible to infer directional dependence based on the proportion of total variance of V that can be explained by the copula regression $r_{V|U}(u)$ compared to the proportion of total variance of U that can be explained by the copula regression $r_{U|V}(v)$ (Sungur 2005; Lee and Kim 2019). We use the method of Lee and Kim 2018 (Lee and Kim 2019) to infer the flow of information for pairwise relationships that

showed a significant relationship in partial correlation analyses. Analyses were performed for allele-specific and total counts and at different developmental time points (excluding 2-4hr and sex chromosomes). X Chromosome data was kept for both analyses and it removal did not change the direction of findings but did increase effect size.

Locus-specific test of gene expression with numbers of open chromatin regions

ATAC-seq peaks were associated to genes at +-1500bp from the TSS and the relationship between the regulatory datasets was tested on a locus-specific basis. We note that the results obtained from this analysis, showing less imbalance in expression with more local peaks, are unlikely to be due to differences in statistical power, as genes linked to shadow enhancers and multiple peaks are typically more highly expressed (wilcox test, $p < 1.5 \times 10^{-69}$). Hence, there is greater power to detect allelic imbalance.

Cis-trans **analysis**

Statistical method for steady-state assignment of cis/trans regulatory mechanism

For one F1 line (vgn x 399), we use maximum likelihood estimation (MLE) to compare parental and offspring ratios simultaneously to determine whether gene expression, chromatin accessibility, H3K4me and H3K27ac enrichments are influenced by *cis*-, *trans,* conserved or both *cis*- and *trans*- acting effects by modeling read counts in parents using negative binomial distributions and the F1 alleles using beta-binomial distribution. We then find the most likely model for each gene.

For each gene, F0 counts from each strain can be modeled as a negative binomial marginal distribution, while F1 counts were modeled using a beta-binomial distribution where the parameters of the beta distribution modeled the proportional contribution from each allele. For each data type, there were 2 replicates (i) for each F0 strain and 2 replicates (j) for F1 samples. F0 counts for each strain (x_i, and y_i) were assumed to follow negative binomial distributions while F1 counts (n_j), were modeled on an allele-specific basis (z_j) using a beta-binomial distribution:

$$x_i \sim Po(\mu_i),\ y_i \sim Po(v_i), z_j \sim Bi(n_j, p_j) \tag{1}$$

$$\mu_i \sim Ga\left(r, \frac{p_\mu}{1-p_\mu}\right), v_i \sim Ga\left(r, \frac{p_v}{1-p_v}\right), p_j \sim Be(\alpha, \beta) \tag{2}$$

where x_i is formally defined as the count of the variant in the ith vgn F0 line, y_i is the binding intensity of the variant in the ith dgrp399 F0 line, n_j is the number of reads mapping across both allelic variants in the jth F1 hybrid and z_j is the number of reads mapping to the vgn allele in the jth F1 hybrid.

We estimate the dispersion parameter *r* for F0 samples using the 'estimateDispersions' function within 'DESeq2' with local regression fit. *r* was used as the reciprocal of the fitted dispersion value from 'DESeq2'.

We constrained parameter estimation for each distribution based on four different regulatory scenarios and derived maximum likelihood values for each hypothetical case on a site-by-site basis. The four models are described below:

$$Conserved: p_\mu = p_\nu \ and \ \alpha = \beta$$

$$Cis: p_\mu \neq p_\nu \ and \ \frac{\alpha}{\alpha + \beta} = \frac{\frac{p_\mu}{1 - p_\mu}}{\frac{p_\mu}{1 - p_\mu} + \frac{p_v}{1 - p_v}}$$

$$Trans: p_\mu \neq p_\nu \ and \ \alpha = \beta$$

$$Cistrans: p_\mu \neq p_\nu \ and \ \alpha \neq \beta$$

Figure 6c, showing the proportion of features assigned to each category, we presented the maximum likelihood assignment. In subsequent analyses, we limited our analyses to features that showed a BIC difference ≥ 2.

For the *cis* and *trans* assignments, we focused only on autosomal features. This was in part due to the complications of calculating *cis*/*trans* components for two different sets of expected ratios, but also because the difference in expected ratio between the X Chromosome and the autosomes can influence the power to detect allelic imbalance and, thus, influence the assignments of *cis* vs. *trans*, with resulting complications for downstream analyses (e.g. categorical enrichment)

Control for difference in staging between samples

A challenge in assessing *cis* and *trans* proportions during development is the influence of differences in staging between samples, which can induce differences in read counts that will not be reflected in allelic ratios. If these differences stem from genetically based differences in developmental rates, then the classification would reflect genuine *trans* differences.

Environmental variation or differences in sample handling, however, can also lead to developmental shifts. To evaluate this possibility, we looked first to see if genes and features classified for *trans* frequently showed evidence of an increase in $\log_2$ fold-changes between time points. For all regulatory layers, we observed a significant increase in $\log_2$ fold-changes between time points for genes and features classified as *trans* ($p < 2.2 \times 10^{-16}$). However, we see no evidence for a coordinated shift in the parental ratios used to calculate *trans* relative to $\log_2$ fold-changes between time points. Indeed, genes and feature counts that increase during development are equally likely to show higher expression in either the maternal line (vgn) or paternal line (DGRP-399). We thus conclude that while a portion of our observed *trans* effects may result from differences in developmental timing, the majority are likely genetic in origin, as global shifts in developmental staging (*e.g.* from handling errors or differences in collection temperature) would induce clear correlations between $\log_2$ fold-change over development and expression bias towards the more developmentally advanced parent.

Calculating broad sense heritability and coefficient of genetic variation

To avoid potential interaction effects with 'time', we fit a separate model for each time point (all three time points in the case of the chromatin data, and excluding 2-4h in the case of RNA). For each gene/feature, we used the lmer function from lme4 to estimate a random effect for line after applying the vst function in DESeq2 to bring the trait values more closely in line with normality. To evaluate the significance of the resulting fit, the model was compared to a null model consisting only of an intercept using the anova function. FDR values were calculated from the resulting vector of p-values using the qvalue function in R (v3.5.1). Estimated line variances and residual variances were extracted from the model using the 'VarCorr' function. Line variances were treated as proportional to broad-sense heritability (H^2). We calculated the *coefficient of genetic variation* by scaling our estimated 'between-line variances' by the variance stabilized mean read count of each feature such that genetic variation is presented as a percentage deviation from the average of the population (Berney et al. 1975; Garfield et al. 2013). We used the formula below:

$$coefficientofgeneticvariation = 100 \times \frac{\sqrt{linevariance}}{traitmean}$$

In our analysis, H^2 and coefficient of genetic variation were considered meaningful as long as the line-model was significant with an FDR < 0.1.

The result of the above process generated one value per time point for each feature. An alternative is to fit a similar model to the above for all times (excluding 2-4h in the case of RNA) and including a term for 'time'. The resulting distributions for H^2 and coefficient of genetic variation were qualitatively similar. The enrichment calculations were carried out as described above.

Measuring additive vs. non-additive heritability

In the case of additively inherited gene expression (or read counts for any of our measured features), the signal observed in the F1 is expected to be equal to the midpoint (average) of the two parents, while non-additively inherited genes/features should show a significant departure from that midpoint. To formally test for non-additivity, we made use of the standard workflow in DESeq2 with two modifications. First, we set the betaPrior option equal to TRUE. After setting the reference genotype to the F1 (vgn x 399) using the relevel function, we then extracted the results using the 'results' function and the contrast vector c(0,1,-.5, -.5) to contrast the full value of the F1 genotype with ½(vgn + 399). Features with an FDR < .1 were considered as "non-additive".

The link between *trans* variation and gene category

Mirroring our buffering results, genes with more regulatory elements in their vicinity are more likely to be classified as *trans*-acting having more peaks in their immediate regulatory domain (*trans* = 2.58 peaks per gene vs. 1.9, p = 0.00094). Similarly, there is a significant, though modest, enrichment of *trans* influences among TFs and a depletion among metabolic genes, two categories that are strongly distinguished in the complexity of their associated regulatory landscapes (Supplemental Table S9). This appears to have downstream impacts on the heritability of gene expression for different gene categories. Among 80 tested gene categories, DNA-binding TFs ($p = 3 \times 10^{-3}$) and interestingly mitochondrial associated genes ($p = 2 \times 10^{-6}$) stand out as the two gene categories with statistically elevated frequencies of non-additive inheritance (Fisher's exact test; Methods). Thus, while TFs generally show reduced genetic variation among lines and reduced allelic imbalance in gene expression (Supplemental Fig. S4), they are still affected by *trans*-acting variants whose non-additive inheritance reduces the efficacy by which selection can alter gene expression differences among different lines.

The impact of *cis* and *trans* variation on patterns of selection

Genes influenced by both *cis* and *trans* acting variants (*cis-trans*) provide an opportunity to understand patterns of recent selection: In the face of compensatory evolution, *cis* and *trans* acting influences are more likely to work in opposite directions, while directional selection will be more likely to reinforce *cis* and *trans* effects acting in the same direction. Using the classification of *cis* and *trans* by Goncalves et al (Goncalves et al. 2012)(see below), we observed that *cis* and *trans* effects were much more likely to act in a compensatory manner as compared to gene expression: for chromatin accessibility and histone modifications, 13% of *cis-trans* features were classified as *same* vs. 37% for RNA (Fig. 6c: $p < 2.2 \times 10^{-16}$ chi^2). This suggests that for RNA there is either more frequent directional selection or less efficient selection against directional changes. This result is robust to the method used to classify *cis* + *trans* effects (Landry et al. 2005), with 63% of *cistrans* RNA features being classified as divergent for RNA vs. 22% for chromatin features (Supplemental Fig. S9d: $p < 2.2 \times 10^{-16}$; chi^2). Taken together, these results suggest clear differences in evolutionary trajectory between regulatory layers which reflects population processes operating at different levels of organization, as well as differences between functional gene classes. The classification of Goncalves et al. (Goncalves et al. 2012) is as follows. For all genes, we asked if their *cis* and *trans* contributes act to reinforce one another (same direction) or if they operated in opposite directions. Formally, for the i_th gene, we define the average $\log_2$ fold change for the parental lines as x_i and the average $\log_2$ allelic ratio from the F1 data as y_i. We then classified:

Opposite – cis stronger: (0 < y_i < x_i) OR (0 > y_i > x_i)

Opposite – trans stronger: (x_i < 0 < y_i) OR (y_i < 0 < x_i)

Same – cis stronger (0 < x_i < y_i < 2x_i) OR (0 > x_i > y_i > 2x_i)

Same – trans stronger (0 < 2x_i < y_i) OR (0 > 2x_i > y_i)

References

Bates D, Mächler, M., Bolker, B., Walker, S. 2015. Fitting Linear Mixed-Effects Models Using lme4. *Journal of Statistical Software* **67**: 48.

Benjamini Y, and Hochberg, Y. 1995. Controlling the False Discovery Rate: A Practical and Powerful Approach to Multiple Testing. *Journal of the Royal Statistical Society: Series B (Methodological)* **57**: 12.

Berney D, Petcher TJ, Schmutz J, Weber HP, White TG. 1975. Conformations and biological properties of apomorphine and its phenanthro(10,1-b,c)azepine homologue. *Experientia* **31**: 1327-1328.

Bourgon R, Gentleman R, Huber W. 2010. Independent filtering increases detection power for high-throughput experiments. *Proc Natl Acad Sci U S A* **107**: 9546-9551.

Campos-Ortega JA, Hartenstein, Volker. 1997. *The Embryonic Development of Drosophila melanogaster*. Springer-Verlag Berlin Heidelberg.

Dobin A, Davis CA, Schlesinger F, Drenkow J, Zaleski C, Jha S, Batut P, Chaisson M, Gingeras TR. 2013. STAR: ultrafast universal RNA-seq aligner. *Bioinformatics* **29**: 15-21.

Garfield DA, Runcie DE, Babbitt CC, Haygood R, Nielsen WJ, Wray GA. 2013. The impact of gene expression variation on the robustness and evolvability of a developmental gene regulatory network. *PLoS Biol* **11**: e1001696.

Ghavi-Helm Y, Jankowski A, Meiers S, Viales RR, Korbel JO, Furlong EEM. 2019. Highly rearranged chromosomes reveal uncoupling between genome topology and gene expression. *Nat Genet* doi:10.1038/s41588-019-0462-3.

Graveley BR, Brooks AN, Carlson JW, Duff MO, Landolin JM, Yang L, Artieri CG, van Baren MJ, Boley N, Booth BW et al. 2011. The developmental transcriptome of Drosophila melanogaster. *Nature* **471**: 473-479.

Hu Y, Comjean A, Perkins LA, Perrimon N, Mohr SE. 2015. GLAD: an Online Database of Gene List Annotation for Drosophila. *J Genomics* **3**: 75-81.

Imrichova H, Hulselmans G, Atak ZK, Potier D, Aerts S. 2015. i-cisTarget 2015 update: generalized cis-regulatory enrichment analysis in human, mouse and fly. *Nucleic Acids Res* **43**: W57-64.

John S, Sabo PJ, Thurman RE, Sung MH, Biddie SC, Johnson TA, Hager GL, Stamatoyannopoulos JA. 2011. Chromatin accessibility pre-determines glucocorticoid receptor binding patterns. *Nat Genet* **43**: 264-268.

Kim J-M, Hwang, S.Y. 2017. Directional dependence via Gaussian copula beta regression model with asymmetric GARCH marginals. *Communications in Statistics - Simulation and Computation* **46**: 15.

Landry CR, Wittkopp PJ, Taubes CH, Ranz JM, Clark AG, Hartl DL. 2005. Compensatory cis-trans evolution and the dysregulation of gene expression in interspecific hybrids of Drosophila. *Genetics* **171**: 1813-1822.

Lara-Astiaso D, Weiner A, Lorenzo-Vivas E, Zaretsky I, Jaitin DA, David E, Keren-Shaul H, Mildner A, Winter D, Jung S et al. 2014. Immunogenetics. Chromatin state dynamics during blood formation. *Science* **345**: 943-949.

Lee N, Kim JM. 2019. Copula directional dependence for inference and statistical analysis of whole-brain connectivity from fMRI data. *Brain Behav* **9**: e01191.

Li H, Durbin R. 2010. Fast and accurate long-read alignment with Burrows-Wheeler transform. *Bioinformatics* **26**: 589-595.

Li H, Handsaker B, Wysoker A, Fennell T, Ruan J, Homer N, Marth G, Abecasis G, Durbin R, Genome Project Data Processing S. 2009. The Sequence Alignment/Map format and SAMtools. *Bioinformatics* **25**: 2078-2079.

Love MI, Huber W, Anders S. 2014. Moderated estimation of fold change and dispersion for RNA-seq data with DESeq2. *Genome Biol* **15**: 550.

Mi H, Vandergriff J, Campbell M, Narechania A, Majoros W, Lewis S, Thomas PD, Ashburner M. 2003. Assessment of genome-wide protein function classification for Drosophila melanogaster. *Genome Res* **13**: 2118-2128.

Nguyen NTT, Contreras-Moreira B, Castro-Mondragon JA, Santana-Garcia W, Ossio R, Robles-Espinoza CD, Bahin M, Collombet S, Vincens P, Thieffry D et al. 2018. RSAT 2018: regulatory sequence analysis tools 20th anniversary. *Nucleic Acids Res* **46**: W209-W214.

Opgen-Rhein R, Strimmer K. 2007. From correlation to causation networks: a simple approximate learning algorithm and its application to high-dimensional plant gene expression data. *BMC Syst Biol* **1**: 37.

Quinlan AR, Hall IM. 2010. BEDTools: a flexible suite of utilities for comparing genomic features. *Bioinformatics* **26**: 841-842differential expression analysis of digital gene expression data. *Bioinformatics* **26**: 139-140.

Sisson JC. 2007. Maintaining a population of Drosophila. *CSH Protoc* **2007**: pdb prot4707.

Skelly DA, Johansson M, Madeoy J, Wakefield J, Akey JM. 2011. A powerful and flexible statistical framework for testing hypotheses of allele-specific gene expression from RNA-seq data. *Genome Res* **21**: 1728-1737.

Sklar A. 1973. Random variables, joint distribution functions, and copulas. *Kybernetika* **9**: 12.

Sungur EA. 2005. A Note on Directional Dependence in Regression Setting. *Communications in Statistics - Theory and Methods* **34**: 9.

Thomsen S, Anders S, Janga SC, Huber W, Alonso CR. 2010. Genome-wide analysis of mRNA decay patterns during early Drosophila development. *Genome Biol* **11**: R93.

Turner LM, Chuong EB, Hoekstra HE. 2008. Comparative analysis of testis protein evolution in rodents. *Genetics* **179**: 2075-2089.

Zhang Y, Liu T, Meyer CA, Eeckhoute J, Johnson DS, Bernstein BE, Nusbaum C, Myers RM, Brown M, Li W et al. 2008. Model-based analysis of ChIP-Seq (MACS). *Genome Biol* **9**: R137.
